# How feasible is nutrition intervention research in eating disorders? Lessons learnt from a pilot parallel randomised controlled trial of tyrosine supplementation in adolescents with anorexia nervosa

**DOI:** 10.1186/s40337-024-01134-5

**Published:** 2024-11-15

**Authors:** Melissa Hart, David Sibbritt, Bridget Wilcken, Lauren T. Williams, Wayne Levick, Kenneth P. Nunn

**Affiliations:** 1 University of Newcastle, Callaghan, NSW 2308 Australia; 2 Hunter New England Mental Health Service, Waratah, NSW 2298 Australia; 3 University of Technology, Ultimo, NSW 2007 Australia; 4 University of Sydney, Camperdown, NSW 2006 Australia; 5 Griffith University, Southport, QLD 4125 Australia

**Keywords:** Nutrition, Tyrosine, Adolescent, Anorexia nervosa, Pilot, Randomised controlled trial

## Abstract

**Objective:**

Eating disorders are complex illnesses with high morbidity and mortality. Yet, there is promising evidence to support the effects of nutrition on the brain and behaviour. One proposed example is the use of tyrosine as an adjunct treatment in anorexia nervosa (AN). However, recruitment and retention in eating disorder clinical trials has posed difficulties for researchers. The aim of this study was to pilot test a parallel randomised controlled trial (RCT) of tyrosine supplementation to explore the feasibility of recruitment and retention, intervention adherence and data collection methods from the perspective of participants and researchers.

**Method:**

Feasibility was assessed using numbers participating, questionnaire completion in patients and parent/carers completing and declining participation, a researcher implementation record and clinical measures. Subjects included adolescents aged 12–17 years with AN. The study was conducted over a 12-week period, with the intervention group receiving 5 mg of L-tyrosine supplement and the control group receiving a placebo.

**Results:**

Recruitment targets were not met and recruitment to a full RCT based on the current study protocol and recruitment sites did not prove feasible. Of the 39 approached for RCT participation, seven were recruited to the RCT (18% response rate) despite extending recruitment periods, with 100% retained and analysed. Patients or parents/carers identified barriers to study participation including burden, the need to consume tyrosine as tablets, and the use of blood, urine and psychological testing. Blood tyrosine rose markedly for subjects in the intervention group. No side effects were reported or measured.

**Conclusions:**

This study offers a unique exploration of the feasibility of a tyrosine trial in anorexia nervosa and is of relevance to assist the success of future nutrition trials. Exploring the suitability of future study designs for nutrition intervention research is warranted.

**Supplementary Information:**

The online version contains supplementary material available at 10.1186/s40337-024-01134-5.

## Abstract (plain language summary)

Eating disorders are serious mental illnesses and exploration of effective treatments are required as a priority. This study explored whether using of a dietary protein (tyrosine) in supplement form added to usual treatment appeared feasible and acceptable to study participants. Study components perceived as potential barriers included use of tyrosine capsules as well as physical and psychological testing. Exploring suitable future study designs for nutrition intervention research with people with an eating disorder and parents/carers is required.

**Supplementary Information:**

The online version contains supplementary material available at 10.1186/s40337-024-01134-5.

## Background

Eating disorders are complex mental illnesses associated with high morbidity and mortality. There is a pressing need to develop and test novel interventions, including nutritional interventions, to modify causal factors and improve treatments for people with an eating disorder. Anorexia nervosa (AN) is an eating disorder with particularly high risk of mortality and a lifetime prevalence of 0.3–4.3% [1]. Onset is often during adolescence and the initial years after onset appear crucial for successful intervention. While the precise aetiology of AN remains unclear, there is growing evidence that neurobiological vulnerabilities contribute [2–5]. One explanatory model suggests that brain noradrenergic dysregulation may be a contributing factor [6].

Noradrenaline is a neurotransmitter linked to feeding and eating behaviour, executive function, attentional state, memory consolidation, neuroplasticity and inhibition of inappropriate thoughts and behaviours, all of which may be altered in people with AN [6–8]. Tyrosine is a dietary amino acid and a key precursor to synthesis of the catecholamines dopamine, noradrenaline and adrenaline [9]. Although catecholamine synthesis is regulated by tyrosine hydroxylase, tyrosine ingestion increases plasma tyrosine, which can subsequently elevate brain tyrosine and increase the synthesis of dopamine and noradrenalin [10]. Tyrosine is derived directly from the diet, from hydrolysis of tissue proteins or from hydroxylation of phenylalanine [11]. Dietary protein intake is often restricted in people with AN and starvation may influence circulating tyrosine concentrations [2, 12]. There has been some suggestion that the amino acid profile and metabolism in AN may be altered [13–15] and different levels of dietary restriction may have different effects on noradrenaline levels [16]. Metabolic consequences of growth processes may further complicate tyrosine metabolism [17]. A systematic review has explored the role of the noradrenergic system in eating disorders and highlighted involvement of the noradrenergic pathways in binge-like behaviors and the potential efficacy of noradrenaline-modulating therapies in the treatment of bulimia nervosa and binge eating disorder [18].

Given the need for novel interventions, our research team proposed a hypothesis that ongoing administration of tyrosine may lessen noradrenergic dysregulation and alleviate clinical changes found in AN [19]. A pharmacokinetics study was then conducted to assess the biological response to an intervention based on tyrosine supplementation. The twelve-week study administered 5 g/day of tyrosine supplements in two adolescents with AN and found individual variation in blood tyrosine response and no reported side effects of the supplementation protocol [20]. It remains unclear, however, whether tyrosine is an effective adjunct to treatment in AN and whether factors such as age, gender, diagnosis, dosage and nutritional intake confound clinical effects. The outcomes of tyrosine supplementation should be tested in a randomised controlled trial (RCT) design. However, given the novelty of the proposed intervention, and observed difficulties with clinical trial recruitment and retention for patients with AN [21], it is important to pilot-test the design to assess feasibility for a full RCT. The aim of this study was to pilot test a parallel RCT of tyrosine supplementation to explore the feasibility of recruitment and retention, intervention adherence and data collection methods from the perspective of participants and researchers [22].

## Methods

The pilot parallel RCT was approved by the Hunter New England Health (reference number 06/05/24/3.06) and University of Newcastle (approval number H-385-0207) Human Research Ethics Committees. A Notification of Intent to Supply Unapproved Therapeutic Goods under the Clinical Trial Notification Scheme was completed and the study was registered with the Australian New Zealand Clinical Trials Registry (ACTRN12609000007235). All participants (including patients and parents/carers) provided signed consent. Feasibility of the RCT design was assessed according to Table 1.

**Table 1:** Feasibility assessment of components of the RCT design

Feasibility objective	Data collection procedure	Analysis method
1. Recruitment and retention	Numbers participating	• Attainment of recruitment target • Recruitment and retention flowchart
	Participant questionnaire	• Differences in characteristics and responses between participants and non-participants
	Researcher implementation record	• Observed feedback from participants or staff regarding recruitment and retention
2. Intervention acceptability, adherence and safety	Participant questionnaire	• Differences between participants and non-participants regarding acceptability of the intervention • Qualitative feedback regarding the intervention
	Researcher implementation record	• Observed feedback from participants or staff regarding the intervention
	Return of unused study supplements	• Unused supplement count
	Physical, psychological and dietary testing	• Physical or psychological changes over the study period (Table 2) • Dietary intake of tyrosine and phenylalanine
3. Procedures for collecting outcome measures	Participant questionnaire	• Differences between participants and non-participants regarding acceptability of the outcome measures • Qualitative feedback regarding the outcome measures
	Researcher implementation record	• Observed feedback from participants or staff regarding outcome measures
	Completion of study assessments and appointments	• Number of study assessments and appointments completed

### Objective 1: Feasibility of recruitment and retention

Eligibility criteria initially included 12–17-year-old females referred to a tertiary paediatric inpatient ward or child and adolescent mental health inpatient unit with an Eating Disorders Examination-generated DSM-IV diagnosis of AN, with < 85% weight for height. Exclusion criteria included use of amino acid supplements within 12 weeks, medical instability, severe medical or neurological illness, phenylketonuria, drug or alcohol abuse within six months or requiring noradrenergic, combined noradrenergic or stimulant medication. Following recruitment difficulties, eligibility was expanded to include males, < 95% weight for height, participants referred to one of three child and adolescent mental health service (CAMHS) community teams within the same health service jurisdiction, and participants requiring selective serotonin reuptake inhibitors or antipsychotics (including Olanzapine), even though secondary effects on associated neurotransmitter systems could not be entirely ruled out.

Potential patients and their parents/carers were approached for participation by treating clinicians involved in their care. Initially four inpatient treating paediatricians or psychiatrists approached patients for participation in the study. After expanding inclusion criteria an additional 26 multidisciplinary CAMHS clinicians approached participants. All clinicians approaching participants were given initial training and follow up review sessions regarding the recruitment procedures and processes.

Random permuted blocks of four were used to randomise participants. This ensured treatment arms remained balanced between inpatient and community recruitment at equally spaced intervals throughout the study. An independent statistician prepared the randomisation list. Participants, researchers and clinicians remained blinded to treatment assignment until completion of data analysis. A recruitment target to estimate clinically meaningful change in Eating Disorders Examination (child version) subscales was set at 34 participants (17 per arm), based on α = 0.05 and power = 80% to detect a 0.10 expected change [23, 24].

Retention strategies included fortnightly phone calls by the first author to participants and parents/carers to enquire about compliance with study supplements and to discuss their questions or concerns. Study appointment times were agreed at the beginning of the trial and reminder phone calls were given the week and the day before appointment times. Recruitment occurred from May 2009 to November 2011.

Study acceptability for patients and parents/carers was assessed using a self-completed questionnaire purpose-developed by the researchers and adapted from a validated treatment evaluation questionnaire by Kelley and colleagues (1989) [25] (see Supplementary Material 1). Eligible participants were invited to anonymously complete the acceptability questionnaire regardless of whether they were participating in the RCT or not. All participants completed the same 17 Likert scale questions and one open-ended item regarding perceived acceptability of study measures and the intervention supplement. Participants completing the RCT also completed Likert scale and open-ended questions regarding noticeable side effects, benefits, other perceived aspects of participation and other nutritional supplement use. Participants declining were also asked two open-ended questions regarding reasons for declining. The questionnaire took approximately ten minutes to complete and was completed either at the time of declining initial participation or on completion of the RCT. Baseline participant characteristics for those completing study questionnaires (those participating and declining RCT) were collected, including age, gender, length of illness, diagnoses, medications and demographics. An implementation record was kept by one of the researchers, including any perceived difficulties or benefits reported by patients, parents/carers or staff.

### Objective 2: Intervention acceptability, adherence and safety

Supplements were produced in white-coloured, unmarked capsules containing 500 mg of L-tyrosine or placebo (microcrystalline cellulose) and administered as two 2.5 g daily doses in addition to treatment as usual (inpatient refeeding or community team family-based treatment). A research pharmacist dispensed supplements according to a pre-determined randomisation list. Nursing staff administered supplements to participants during hospital admissions. Parents/carers supervised supplement administration to participants in the community. Participants returned unused supplements at weeks six and 12. As this was a pilot study, a broad range of outcome measures were included to assist in exploring clinical effects and determining suitability of outcome measures for future studies (Table 2).

**Table 2:** Physical, psychological and dietary measures used in the study

Test type	Outcome measured	Test Frequency	Test used
Physical	% expected body weight	Baseline and weeks six and 12	Height and weight
	Blood tyrosine	Baseline and weeks one, six and 12	Venepuncture and blood spot
	Urinary catecholamines and metabolites	Baseline and weeks one, six and 12	Urine sample
Psychological	Eating disorders psychopathology	Baseline and week 12	Eating Disorders Examination Child Version (ChEDE) [24]
	Anxiety	Baseline and weeks one, six and 12	State-Trait Anxiety Inventory Form Y-1 at [26]
	Depression	Baseline and weeks six and 12	Children’s Depression Inventory [27]
	Obsessive compulsive symptoms	Baseline and weeks six and 12	Children’s Obsessive Compulsive Inventory [28]
	General mental health	Baseline and weeks six and 12	Strengths and Difficulties Questionnaire (SDQ) [29].
	Cognitive Function	Baseline and week 12	Standardised versions of the Rey Complex Figure Test copy and 30 min recall [30], Verbal Fluency [31], Tower Task [31], Color − Word Interference [31], Verbal Paired Associate Learning [32], Digit Symbol-Coding [33], Visual Learning [34], Matching [35], Trail Making [31], Design Fluency [31] and Reading [36].
Dietary	Dietary tyrosine and phenylalanine intake	Week one, six and 12	24-hour dietary recall [37]

Note: height and weight were taken by trained paediatric nursing staff; % expected body weight was based on 100% weight for height being the 50th percentile Body Mass Index (BMI) [38]; bloods were collected via venepuncture and blood spot at time 0 (supplement ingestion) and two hours post-supplement ingestion; due to resource issues only blood spot samples were analysed for tyrosine using tandem mass spectrometry; urine samples were collected four hours after supplement administration and analysed using high performance liquid chromatography with electrochemical detection; as the intervention may impact trait anxiety, the STAI was administered at four time points; a trained neuropsychologist blinded to treatment assignment conducted cognitive function testing; Reliable Change Index (RCI) methods were used to examine change in psychological tests [39]. For cognitive function tests, both the RCI adjusted for practice effects method by Chelune and colleagues [40] and the regression-based change formula by Maassen and colleagues [41] were selected. A cut-off for significant reliable change was defined as +/-1.645 [42]

### Objective 3: Procedures for collecting outcome measures

Feasibility of the procedures for collecting outcome measures was assessed by participant questionnaire items relating to procedures, researcher implementation records and completion of study assessments and appointments.

### Statistical Analysis and Progression Criteria

Descriptive analysis was used to assess recruitment, retention, intervention components and data collection methods. Open-ended questions from acceptability questionnaires were analysed descriptively. Themes arising from the researcher record of implementation and reflections were grouped and reported. Statistical testing was not conducted due to inadequate sample size.

## Results

### Objective 1: Feasibility of recruitment and retention

**Fig. 1:**
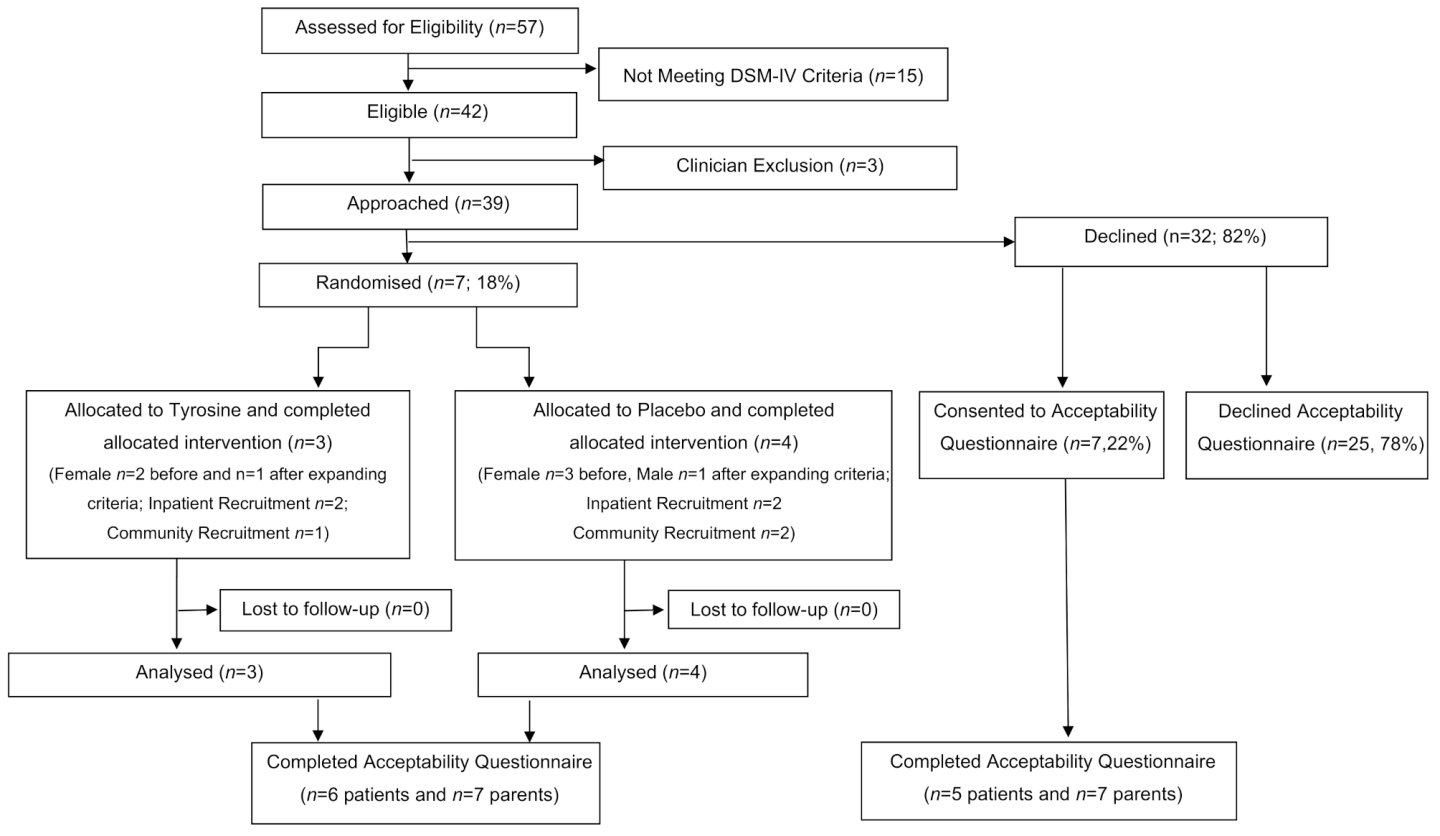
Patient recruitment and retention flowchart for the tyrosine pilot randomised controlled trial Note: DSM-IV denotes Diagnostic and Statistical Manual of Mental Disorders, 4th Edition

Of the fifty-seven patients referred to the recruitment sites with AN during the recruitment period, 42 were eligible to participate and 15 did not meet the necessary DSM-IV criteria upon full assessment (Fig. 1). A treating clinician felt three of the 42 patients were inappropriate to participate due to high burden already experienced by the patient and family, a chaotic home situation meaning the family may not be able to participate, or the patient not aligning with existing treatment or taking medications, and were not approached. Expanding inclusion criteria due to low recruitment resulted in recruitment of two additional participants (one female on psychiatric medications and one male at 89% expected body weight). Seven eligible participants approached for participation agreed to participate in the pilot RCT (18% consent rate) and were randomly allocated to tyrosine (*n* = 3) and placebo (*n* = 4). In addition, seven patients (22%) declining RCT participation agreed to participation in the acceptability questionnaire. Staff at one community team raised reluctance to approach patients in case taking tablets was viewed as an easier treatment option and encouraged drop-out from therapy. Retention for the RCT study was 100%, with no loss to follow up.

The median age of participants consenting and declining RCT participation was 15 (range 12–17) and median length of illness was 1 year for both groups. All were female, aside from one male in the RCT. Those consenting had a higher median percentage expected body weight (84%) compared to those declining (79%). A higher portion of patients consenting had at least one parent with a tertiary education (57% compared to 29%) and lower parent unemployment (0% compared to 43%).

Most patients (73%, *n* = 8) and parents/carers (93%, *n* = 13) reported the study to be acceptable overall (Supplementary Material 2, Table 6). Although most parents (71%, *n* = 10) agreed or strongly agreed to have a positive reaction to the study, only half of patients consenting (*n* = 3, 50%) and one patient declining (20%) experienced a positive reaction. Most patients (*n* = 7; 64%) and parents/carers (*n* = 10; 71%) identified the study would be an additional worry. One parent/carer stated “*it was yet another thing on her plate…would have further increased her stress levels in dealing with her anorexia and all it entails*”.

There was only one parent and there were no patients who believed the study would result in permanent improvement, although many reported benefits of involvement in the RCT (Supplementary Material 2, Table 6). Patients in the RCT cited helping others (*n* = 1), increasing their understanding of their illness (*n* = 3) and the potential for helping their illness (*n* = 2) as beneficial, for example, “…*trying to find a way to help recovery*” and “*I know more about what thoughts worry me more*”. Parents/carers in the RCT cited helping other children or parents faced with the illness (*n* = 6) and having more information about their child’s illness (*n* = 1) as beneficial, for example “*if it can help another child or parent deal with anorexia…has got to be beneficial*”, “*It gave me hope that perhaps down the track some form of help would be available to persons with anorexia*” and “*we need more information about possible ways to approach healing for this terrible disease*”. Insights from researcher reflections included some patients expressing a keen interest in the science behind the study and in helping others. Some patients appeared to dislike use of the term anorexia nervosa.

Other recruitment difficulties were encountered including significant service changes in the major recruiting ward, resulting in pauses in recruitment and a reduction in the potential pool of participants. To counter this the research team expanded inclusion criteria, prolonged recruitment timeframes and increased the number of recruitment sites. Variation in study interest by recruitment staff was noted.

### Objective 2: Intervention acceptability, adherence and safety

Using a form of nutrition as an intervention was found to be acceptable by most parents/carers (86%, *n* = 12), and patients consenting (67%, *n* = 4), though only two patients declining (40%) found using nutrition was acceptable (Supplementary Material 2, Table 6). There were only five parents/carers (36%), two patients consenting (33%) and no patients declining (0%) who found using tablets acceptable. Belief in potential side effects was raised by three patients (*n* = 1 consenting and *n* = 2 declining) and concern for the effects of supplementation by four patients (*n* = 2 consenting and *n* = 2 declining). One patient stated “*when I am taking tablets I feel like I’m being drugged*”. Patients reported concern for the number of tablets required, the potential for weight gain and timing of supplement ingestion. One parent stated concern their child “*would be taking something that was manufactured and under research/experimentation*”. Some patients and parents/carers requested tyrosine be given as opposed to placebo (avoiding randomisation), and questions included side effects and whether tyrosine would effect the brain. There were no side effects of taking study supplements reported by participants, parents/carers or staff. One patient on tyrosine deteriorated towards the end of the study and did not complete final psychological testing (analysed according to intention-to-treat).

Table 3 below shows patient characteristics, study supplement dosage, and estimated daily dietary macronutrient and large neutral amino acid intakes during the study. Unused supplement count for those in the tyrosine group was 7% and for the placebo group 6%. The median tyrosine participant dosage was 104 mg/kg (range 99–122 mg/kg). In addition to the 5 g/day supplement dose, median dietary tyrosine intake was 3–4 g/day and phenylalanine 4–5 g/day.

**Table 3:** Patient characteristics, daily supplemental dosage, and estimated daily dietary macronutrient and large neutral amino acid intakes during the study (*n* = 7)

Item	Tyrosine *(n* = 3)	Placebo (*n* = 4)
	**Median (Range)**	**Median (Range)**
Baseline age (years)	15 (12–17)	15 (15–17)
Baseline length of illness (months)	18 (3–24)	9 (5–36)
Baseline body weight (kg)	48.7 (39.6–48.9)	44.5 (42.7–57.0)
Baseline % expected body weight	85 (84–91)	81 (80–89)
	**Frequency (%)**	**Frequency (%)**
AN Treatment Site		
Inpatient and Community	3 (100)	3 (75)
Community Only	0 (0)	1 (25)
Psychiatric Medications		
Olanzapine, SSRI and sedative	1 (33)	0 (0)
Olanzapine Only	1 (33)	1 (25)
SSRI Only	0 (0)	1 (25)
Vitamin/Mineral Supplement Use	1 (33)	2 (50)
NGF or Oral Nutritional Supplement	1 (33)	1 (33)
	**Median (Range)**	**Median (Range)**
Study Supplements Dosage	5 g/day (10 capsules) 104 mg/kg (99–122 mg/kg)	5 g/day (10 capsules) 108 mg/kg (86–118 mg/kg)
Unused Supplements (500 mg capsules)	62 (27–150) (7%)	54 (23–215) (6%)
Dietary Energy (kJ)	9927 (9603-10,065)	9194 (5328-14,484)
Dietary Fat (g)	84 (79–102)	83 (62–171)
Dietary Protein (g)	114 (83–117)	94 (47–137)
Dietary Carbohydrate (g)	282 (255–291)	258 (133–339)
Dietary Fibre (g)	23 (10–34)	21 (6–34)
Dietary Fluid (g)	1699 (1383–2247)	1861 (1306–2196)
Dietary Phenylalanine (g)	5.0 (3.5–5.2)	4.0 (1.7–8.3)
Dietary Tryptophan (g)	1.3 (0.9–1.5)	1.0 (0.5–2.2)
Dietary Leucine (g)	8.3 (5.8–10.2)	6.6 (3.1–15.0)
Dietary Isoleucine (g)	5.0 (3.5–5.5)	3.9 (1.8-9.0)
Dietary Valine (g)	5.7 (4.1–6.6)	4.6 (2–10)
Dietary Methionine (g)	2.3 (1.5–2.9)	1.7 (0.8–4.5)
Dietary Tyrosine (g)	4.0 (2.8-5.0)	3.3 (1.5–7.6)

Note: AN denotes anorexia nervosa; Olanzapine is an antipsychotic medication; SSRI denotes selective serotonin reuptake inhibitor (an antidepressant); and NGF denotes naso-gastric feed. Median dietary intake in addition to study supplement use is shown in the table above

At baseline, both groups showed normal pre-load blood tyrosine (35–108µmol/L), which rose markedly two hours post-supplementation in tyrosine participants (137–260µmol/L) (Table 4). The magnitude of change in blood tyrosine response to supplementation reduced over time in the tyrosine group (352% baseline to 59% week 12). There were no clinically significant differences detected for change in urinary catecholamines or metabolites.

**Table 4:** Comparison of the Median Group Blood Tyrosine Response (µmol/L) to Supplementation Over Time (*n* = 7)

	Blood Tyrosine Time 1 (0 h) Median (Range)	Blood Tyrosine Time 2 (2 h) Median (Range)	Absolute Difference (% Change)
Baseline			
Tyrosine (*n* = 3)	48 (35–80)	217 (137–260) †	169 (352%)
Placebo (*n* = 4)	65 (40–108)	66 (40–113)	1 (2%)
Week 1			
Tyrosine (*n* = 3)	54 (54–149)	186 (153–249) †	132 (244%)
Placebo (*n* = 4)	50 (39–94)	55 (31–107)	5 (10%)
Week 6			
Tyrosine (*n* = 3)	69 (68–99)	136 (107–291) †	67 (97%)
Placebo (*n* = 4)	70 (40–111)	76 (45–106)	6 (9%)
Week 12			
Tyrosine (*n* = 3)	71 (67–84)	113 (93–264) †	42 (59%)
Placebo (*n* = 4)	52 (49–77)	47 (23–97)	-5 (-10%)

Note: Time 1 denotes time of supplement ingestion, Time 2 denotes 2 h post-supplement ingestion; Absolute Difference denotes the group difference in Time 1 and Time 2 median blood tyrosine; % Change denotes percentage change in group Time 1 and Time 2 median blood tyrosine; and † denotes outside of the laboratory reference range

The tyrosine group showed improvement by week 12 in overall eating disorders psychopathology (chEDE Global Score RCI=-2.89), Eating Concern (RCI=-2.36), Weight Concern (RCI=-2.93) and Shape Concern (RCI=-2.29) (Table 5). The placebo group showed improvement in Eating Concern (RCI=-2.12). By week 12, median percent expected body weight had increased by 2% (to 87%) in the tyrosine group and 6% (to 87%) in the placebo group. Descriptive data for the remainder of the physical and psychological testing are shown in Supplementary Material 2.

**Table 5:** Comparison of median group change in eating disorders psychopathology (chEDE) following twelve weeks of supplementation (tyrosine *n* = 3; placebo *n* = 4)

Scale	Baseline (*n* = 7) Median (Range)	Week 12 (*n* = 6) Median (Range)	Absolute Difference (% Change)	RCI
**Restraint**				
Tyrosine	3.6 (1.4–4.8) †	3.3 (3.0-3.6) †	-0.3 (-8%)	-0.27
Placebo	4.1 (3.2-6.0) †	2.9 (1.2–5.8) †	-1.2 (-29%)	-1.07
**Eating Concern**				
Tyrosine	3.2 (1.4–3.6) †	2.2 (1.2–3.2) †	-1.0 (-31%)	-2.36*
Placebo	3.9 (2.8-5.0) †	3.0 (1.8–4.4) †	-0.9 (-23%)	-2.12*
**Weight Concern**				
Tyrosine	5.0 (3.0-5.4) †	2.8 (2.2–3.4) †	-2.2 (-44%)	-2.93*
Placebo	4.8 (3.8-5.0) †	3.8 (2.6–5.4) †	-1.0 (-21%)	-1.33
**Shape Concern**				
Tyrosine	4.5 (3.1–4.9) †	2.5 (1.8–3.3) †	-2.0 (-44%)	-2.29*
Placebo	5.5 (5.4–5.6) †	4.9 (3.9–5.3) †	-0.6 (-11%)	-0.69
**Global Score**				
Tyrosine	4.3 (2.2–4.5) †	2.7 (2.0-3.4) †	-1.6 (-37%)	-2.89*
Placebo	4.4 (4.2–5.4) †	3.7 (2.5–5.1) †	-0.7 (-16%)	-1.26

Note: chEDE denoted Eating Disorders Examination, child version, questions are rated on a 7-point scale with higher scores representing greater psychopathology; % Change denotes percentage change in group median raw score over time; Absolute Difference denotes the difference in group baseline and follow-up median raw scores; RCI denotes reliable change index formula based on Jacobson et al. (1991) [43]; † denotes a mean score within the clinically significant range, based on a z score of two or more (calculated from Wade et al., 2008) [44]; ‡ denotes a clinically significant change (moved into or out of the clinically significant range); and *denotes statistically significant reliable change (RCI = ± 1.645) [42]

### Objective 3: Procedures for collecting outcome measures

All parents/carers consenting (100%, *n* = 7) and most declining (71%, *n* = 5) found study procedures to be acceptable, though only one-third of patients consenting (33%, *n* = 2) and no patients declining participation (0%, *n* = 0) found procedures were acceptable (Supplementary Materials, Table 6). Some parents/carers (*n* = 6; 43%) and most patients (*n* = 7; 64%) believed there would be potential discomfort associated with the study. Use of blood and urine testing was found to be acceptable by only four parents/carers (29%) and no patients (0%). One patient stated “*because I hate blood tests*”. Most parents/carers (*n* = 11; 79%), though only three patients consenting (50%) and no patients declining (0%) found use of psychological tests acceptable. One patient stated “*because I don’t want people asking me how I feel about my weight*”. A researcher reflection was that patients often reported not wanting blood, urine or psychological testing.

All RCT participants attended 100% of required study appointments and assessments aside from one participant unable to complete final testing. It was noted those who chose to participate appeared highly motivated to complete the trial and associated assessments. Overall nursing, medical and allied health staff reported that recruitment procedures were clear, worked well and study implementation ran smoothly.

## Discussion

Building the evidence-base for effective nutrition interventions for people with an eating disorder is a priority, though clinical trials are lacking. This pilot study aimed to explore the feasibility of conducting a definitive RCT of tyrosine administration in AN. Findings from this study highlight important aspects of feasibility and provide key insights into conducting nutrition intervention research in eating disorders. The study is highly relevant to implementation of studies in AN involving nutrition, use of tablets, physical or psychological testing.

Recruitment to a full RCT using the current study protocol and nominated recruitment sites did not prove feasible. Recruitment targets were not reached and proved to be a significant issue. Given the total recruitment time for seven participants took 30 months, it would take approximately 12.5 years to recruit the 34 participants needed. This raises the need to consider key factors in facilitating future nutrition intervention trials in eating disorders. Most patients declined participation in the study and did not have a positive reaction to the study when it was presented to them. This raises the need for future collaborative studies with people with a lived experience of an eating disorder to explore factors that may facilitate or hinder participation in nutrition intervention trials. Consideration of participant burden is important, with most participants identifying the study would be an additional worry. Unanticipated service changes contributed to a reduction in potential participants and the need to extend recruitment sites and time frames. Using multiple treatment sites for shorter periods and including both outpatient and inpatient programs as recruitment sources have been suggested as useful recruitment strategies [45, 46], though did not prove successful in this study. Conducting RCTs in metropolitan, established specialist eating disorder services may minimise service fluctuations, facilitate access to more participants and enable involvement of fewer staff approaching participants within their specialist role. Minimising the number of staff involved in approaching potential participants, minimising the length of recruitment time (retaining staff motivation) and provision of targeted training and ongoing support to staff regarding acceptable presentation of the study to participants are recommended. Retention was not an issue and completion of study assessments proved satisfactory. The study demonstrated the need for a comprehensive recruitment and retention plan, with cut points for ceasing recruitment.

The intervention (tyrosine as a nutritional supplement) proved to be a barrier for participation, which is unsurprising considering the potential discomfort associated with increased nutrition and weight gain in AN. A promising finding was that there was a level of acceptability from both patients and parents/carers towards using a form of nutrition in the study. Use of tablets under experiment and concern for the negative impact of medications were, however, raised as key issues for both patients and parents/carers. This finding is consistent with previous pharmacotherapy trials. Two pharmacotherapy trials in adolescents with AN had found only 21–26% consented to participation, the main reasons for declining were not wanting to initiate medications and concern for potential side effects such as weight gain [21, 47]. Another study in adolescents offering adjunctive pharmacotherapy treatment with fluoxetine in addition to a trial of family therapy interventions, found that removing the medication arm of the trial resulted in improved recruitment [48]. Although adult consent rates in medication trials appear higher 41–68% [49–53], the medication-taking concern expressed by those declining participation remains [51]. Parental involvement may increase adolescent treatment acceptance and attendance [45, 54], although this may not be true for medication trials. In one study, parents of younger patients appeared reluctant to experiment with medications with limited efficacy [48]. Clinician concern for patient participation in medication trials was reported in this study. The desire to avoid randomisation (receive active intervention only) was also raised by participants.

Participants receiving tyrosine showed a marked change in blood tyrosine in response to supplementation, which reduced over the 12 weeks. This change over time could suggest up-regulation at different points in the metabolic pathway, consistent with the hypothesis proposed by Hart and colleagues (2013) [19]. Although there were no reported, observed or measured side effects, this is a small sample and one patient deteriorated during the study. A randomised cross over trial administering tyrosine in adults with AN has been conducted and found improvements in memory tasks and mood, and no side effects [55]. Safety of tyrosine supplementation cannot be concluded from this pilot study. Conclusions from patient outcome measures in this study also cannot be considered due to the very small sample size.

The outcome measures involving collection of blood, urine and psychological tests proved to be problematic and require attention for future studies. Blood testing may need to be minimised in future studies to facilitate acceptability. There were no clinically significant changes in urinary catecholamines or metabolites, hence urine testing could be omitted. Patients raised concerns around the acceptability of psychological testing. This is not surprising considering the potential stigma associated with psychological testing and the difficulty associated with discussing weight, shape and eating for people with AN. This raises difficulties for researchers, however, as the gold standard for assessment of eating disorders psychopathology involves discussing thoughts around weight, shape and eating [56]. Using a shorter psychological assessment battery should be considered. Future studies are required to assist in determining barriers and facilitators to participation in nutrition intervention studies for people with eating disorders. Involving people with eating disorders and parents/carers in identifying participation barriers and designing future nutritional supplementation studies is important. Larger samples are required to determine safety, dosage and effectiveness of using tyrosine in AN, and to account for confounders which may influence study outcomes (including gender, age, dosages, medications, nutritional status, active re-feeding, the effects of food, purging and biological adaption). Comparison of tyrosine to control groups and other treatments is also required.

The key limitation to this study is the small sample size, meaning safety, dosage, clinical effects or estimations of effect sizes could not be explored. Feasibility aspects of this study contributes to the very limited evidence available to guide researchers in progressing studies of nutritional supplementation in eating disorders. The detailed focus on study acceptability provides important insights into participation issues.

## Conclusions

This pilot study offers a unique exploration of the feasibility of a nutritional supplementation (tyrosine) RCT in adolescents with AN. Increasing evidence of the effects of nutrition on the brain and behaviour in AN is of particular interest, along with progressing novel interventions that could modify causal and maintaining factors. The difficulties associated with obtaining high quality evidence, particularly in terms of recruitment and retention, have proven to be a significant barrier to progressing treatment research. This study highlights a range of factors that require attention prior to progressing to a full scale RCT of tyrosine in AN, including suitability of recruitment sites, use of nutrition in a tablet form and use of selected physical and psychological outcome measures.

## Electronic Supplementary Material

Below is the link to the electronic supplementary material.

Supplementary Material 1

Supplementary Material 2

## Data Availability

The datasets generated and/or analysed during the current study are not publicly available due to the small sample size and participant privacy, though are available from the corresponding author on reasonable request.

## Declarations

### Clinical Trial Registration

The study was registered with the Australian New Zealand Clinical Trials Registry (ACTRN12609000007235).

## Abbreviations

AN: Denotes anorexia nervosa
chEDE: Denotes Eating Disorders Examination, child version
EDE: Denotes Eating Disorders Examination
