# Supplementary material for: How feasible is nutrition intervention research in eating disorders? Lessons learnt from a pilot parallel randomised controlled trial of tyrosine supplementation in adolescents with anorexia nervosa
Source: J Eat Disord. 2024 Nov 15;12:181. doi: 10.1186/s40337-024-01134-5 (PMC11568674; doi:10.1186/s40337-024-01134-5)
Supplement: Supplementary file 1 — Supplementary Material 1 [file 40337_2024_1134_MOESM1_ESM.pdf]

How feasible is nutrition intervention research in eating disorders? Lessons learnt from a pilot parallel randomised controlled trial of tyrosine supplementation in adolescents with anorexia nervosa

Supplementary Material 1: Acceptability Questionnaires

Patients Completing the Tyrosine Study

The following questionnaire aims to assist researchers to improve their understanding of the acceptability of this study for people with restrictive eating disorders and their families, and also in what might influence the decision to participate in the study.

The questionnaire consists of a variety of questions. Please attempt to answer every question. You may be asked to write answers to a question, or you may be provided with a scale for recording your response. For all questions with a scale, please place "X" for the answer that most applies to you.

	Strongly Disagree	Disagree	Neither Agree or Disagree	Agree	Strongly Agree
1a.Overall, I found the study to be acceptable	☐	☐	☐	☐	☐
b. I like the procedures used in this study	☐	☐	☐	☐	☐
c. I liked that the study involved using a form of nutrition	☐	☐	☐	☐	☐
d. I liked that the study involved taking tablets	☐	☐	☐	☐	☐
e. I liked that the study involved blood or urine samples	☐	☐	☐	☐	☐
f. I liked that the study involved psychological tests and questionnaires	☐	☐	☐	☐	☐
g. I believe the treatment is likely to be effective	☐	☐	☐	☐	☐
h. I believe that participants involved in the study may experience discomfort	☐	☐	☐	☐	☐
i. I believe that participants involved in the study may experience negative side effects	☐	☐	☐	☐	☐
j. I was worried about the effects the supplements were having on me	☐	☐	☐	☐	☐
k. Being involved in the study meant another thing I had to worry about	☐	☐	☐	☐	☐
	Strongly	Disagree	Neither Agree	Agree	Strongly

	Disagree		or Disagree		Agree
l. I believe the study is likely to result in permanent improvement in my symptoms	☐	☐	☐	☐	☐
m. I find the researcher is knowledgeable about the topic under study and the study procedures	☐	☐	☐	☐	☐
n. I find the researcher is trustworthy	☐	☐	☐	☐	☐
o. Overall I have a positive reaction to this study	☐	☐	☐	☐	☐
p. I believe that it would be acceptable to use this treatment without a patient's consent	☐	☐	☐	☐	☐
q. I believe it would be acceptable to use this study with individuals who cannot choose treatments for themselves	☐	☐	☐	☐	☐

2. If you were worried about the effects the supplements were having on you, please describe the effects you were worried about:

____

____

____

____

3a. Did you experience any noticeable side effects from taking the supplements (eg stomach ache, bad taste in the mouth, felt unwell, reduced appetite, feeling anxious etc)?

Yes ☐ No ☐ (please "X")

b. If yes, please describe the side effects:

____

____

____

____

4a. Could you taste the supplement that you were taking?

Yes ☐ No ☐ (please "X")

b. If yes, please describe what you could taste:

____

____

____

____

5. Try to think about any changes that you have noticed in the way that you were feeling or thinking over the past 3 months (since starting the study) and select the answer below that most applies to you:

	Definitely Worse	Somewhat Worse	No Noticeable Change	Somewhat Better	Definitely Better
a. Your mood	☐	☐	☐	☐	☐
b. Feelings of anxiety or anxiousness	☐	☐	☐	☐	☐
c. Worrying thoughts that you can't stop thinking about	☐	☐	☐	☐	☐
d. Ability to think things through	☐	☐	☐	☐	☐
e. Worry about your weight or shape	☐	☐	☐	☐	☐

6a. Do you feel that being involved in the study was beneficial in any way?

Yes ☐ No ☐ (please "X")

b. If yes, please specify why you thought the study was beneficial:

____

____

____

____

7a. Is there anything else about the study that made it easy or difficult to complete that you have not mentioned?

Yes ☐ No ☐ (please "X")

b. If yes, please specify:

____

____

____

____

8a. Did you use any other nutritional supplement while you were involved in this study?

Yes ☐ No ☐ (please "X")

b. If yes, please specify the name of the supplement and how much you were taking:

____

____

____

____

Thankyou for your time and feedback!

<u>Parent or Carer Completing the Tyrosine Study</u>

The following questionnaire aims to assist researchers to improve their understanding of the acceptability of this study for people with restrictive eating disorders and their families, and also in what might influence the decision to participate in the study.

The questionnaire consists of a variety of questions. Please attempt to answer every question. You may be asked to write answers to a question, or you may be provided with a scale for recording your response. For all questions with a scale, please place "X" for the answer that most applies to you.

	Strongly Disagree	Disagree	Neither Agree or Disagree	Agree	Strongly Agree
1a. Overall, I found the study to be acceptable	☐	☐	☐	☐	☐
b. I like the procedures used in this study	☐	☐	☐	☐	☐
c. I liked that the study involved using a form of nutrition	☐	☐	☐	☐	☐
d. I liked that the study involved taking tablets	☐	☐	☐	☐	☐
e. I liked that the study involved blood or urine samples	☐	☐	☐	☐	☐
f. I liked that the study involved psychological tests and questionnaires	☐	☐	☐	☐	☐
g. I believe the treatment is likely to be effective	☐	☐	☐	☐	☐
h. I believe that participants involved in the study may experience discomfort	☐	☐	☐	☐	☐
i. I believe that participants involved in the study may experience negative side effects	☐	☐	☐	☐	☐
j. I was worried about the effects the supplements were having on my child	☐	☐	☐	☐	☐
k. Being involved in the study meant another thing I had to worry about	☐	☐	☐	☐	☐

	Strongly Disagree	Disagree	Neither Agree or Disagree	Agree	Strongly Agree
l. I believe the study is likely to result in permanent improvement in my child's symptoms	☐	☐	☐	☐	☐
m. I find the researcher is knowledgeable about the topic	☐	☐	☐	☐	☐

under study and the study procedures					
n. I find the researcher is trustworthy	☐	☐	☐	☐	☐
o. Overall I have a positive reaction to this study	☐	☐	☐	☐	☐
p. I believe that it would be acceptable to use this treatment without a patient's consent	☐	☐	☐	☐	☐
q. I believe it would be acceptable to use this study with individuals who cannot choose treatments for themselves	☐	☐	☐	☐	☐

2. If you were worried about the effects the supplements were having on your child, please describe the effects you were worried about:

____

____

____

____

____

3a. Did your child experience any noticeable side effects from taking the supplements (eg stomach ache, bad taste in the mouth, felt unwell, reduced appetite, feeling anxious etc)?

Yes ☐ No ☐ (please "X")

b. If yes, please describe the side effects:

____

____

____

____

____

4. Try to think about any changes that you have noticed in the way that your child has been feeling or thinking over the past 3 months (since starting the study) and select the answer below you feel most applies to your child:

	Definitely Worse	Somewhat Worse	No Noticeable Change	Somewhat Better	Definitely Better
a.Your child's mood	☐	☐	☐	☐	☐
b. Your child's feelings of anxiety or anxiousness	☐	☐	☐	☐	☐
c. Worrying thoughts that your child can't stop thinking about	☐	☐	☐	☐	☐
d. Your child's ability to think things through	☐	☐	☐	☐	☐
e. Your child's worry about their weight or shape	☐	☐	☐	☐	☐

5a. Do you feel that being involved in the study was beneficial in any way?

Yes ☐ No ☐ (please "X")

b. If yes, please specify why you thought the study was beneficial:

____
____
____
____
____

6a. Is there anything else about the study that made it easy or difficult to complete that you have not mentioned?

Yes ☐ No ☐ (please "X")

b. If yes, please specify:

____
____
____
____
____

7a. Did your child use any other nutritional supplement while they were involved in this study?

Yes ☐ No ☐ (please “X”)

b. If yes, please specify the name of the supplement and how much they were taking:

____

____

____

____

____

Thankyou for your time and feedback!

<u>Patient Declining Participation in the Tyrosine Study</u>

There is no obligation to complete this questionnaire. Participation is *entirely* voluntary.

The following questionnaire aims to assist researchers to improve their understanding of the acceptability of this study for people with restrictive eating disorders and their families, and also in what might influence the decision to participate in the study.

The questionnaire consists of a variety of questions. Please attempt to answer every question. You may be asked to write answers to a question, or you may be provided with a scale for recording your response. For all questions with a scale, please place "X" for the answer that most applies to you.

1. What was the main reason for declining participation in this study (please describe)?

____

____

____

____

____

How would you rate the following:

	Strongly Disagree	Disagree	Neither Agree or Disagree	Agree	Strongly Agree
2a.Overall, I found the study to be acceptable	☐	☐	☐	☐	☐
b. I like the procedures used in this study	☐	☐	☐	☐	☐
c. I liked that the study involved using a form of nutrition	☐	☐	☐	☐	☐
d. I liked that the study involved taking tablets	☐	☐	☐	☐	☐
e. I liked that the study involved blood or urine samples	☐	☐	☐	☐	☐
f. I liked that the study involved psychological tests and questionnaires	☐	☐	☐	☐	☐
g. I believe the treatment is likely to be effective	☐	☐	☐	☐	☐

	Strongly Disagree	Disagree	Neither Agree or Disagree	Agree	Strongly Agree
h. I believe that participants involved in the study may experience discomfort	☐	☐	☐	☐	☐

i. I believe that participants involved in the study may experience negative side effects	☐	☐	☐	☐	☐
j. I was worried about the effects the supplements would have on me	☐	☐	☐	☐	☐
k. Being involved in the study meant another thing I would have to worry about	☐	☐	☐	☐	☐
l. I believe the study is likely to result in permanent improvement in my symptoms	☐	☐	☐	☐	☐
m. I find the researcher is knowledgeable about the topic under study and the study procedures	☐	☐	☐	☐	☐
n. I find the researcher is trustworthy	☐	☐	☐	☐	☐
o. Overall I have a positive reaction to this study	☐	☐	☐	☐	☐
p. I believe that it would be acceptable to use this treatment without a patient's consent	☐	☐	☐	☐	☐
q. I believe it would be acceptable to use this study with individuals who cannot choose treatments for themselves	☐	☐	☐	☐	☐

3. If you were worried about the effects the supplements would have on you, please describe the effects you were worried about:

____

____

____

____

4a. Are there any other reasons involved in deciding not to participate in this study that you can think of?

Yes ☐ No ☐ (please "X")

b. If yes, please specify:

____

____

____

____

____

Thankyou for your time and feedback!

Parent or Carer Declining Participation in the Tyrosine Study

There is no obligation to complete this questionnaire. Participation is *entirely* voluntary.

The following questionnaire aims to assist researchers to improve their understanding of the acceptability of this study for people with restrictive eating disorders and their families, and also in what might influence the decision to participate in the study.

The questionnaire consists of a variety of questions. Please attempt to answer every question. You may be asked to write answers to a question, or you may be provided with a scale for recording your response. For all questions with a scale, please place "X" for the answer that most applies to you.

2. What was the main reason for declining participation in this study (please describe)?

____

____

____

____

How would you rate the following:

	Strongly Disagree	Disagree	Neither Agree or Disagree	Agree	Strongly Agree
2a. Overall, I found the study to be acceptable	☐	☐	☐	☐	☐
b. I like the procedures used in this study	☐	☐	☐	☐	☐
c. I liked that the study involved using a form of nutrition	☐	☐	☐	☐	☐
d. I liked that the study involved taking tablets	☐	☐	☐	☐	☐
e. I liked that the study involved blood or urine samples	☐	☐	☐	☐	☐
f. I liked that the study involved psychological tests and questionnaires	☐	☐	☐	☐	☐
g. I believe the treatment is likely to be effective	☐	☐	☐	☐	☐
h. I believe that participants involved in the study may experience discomfort	☐	☐	☐	☐	☐

	Strongly Disagree	Disagree	Neither Agree or Disagree	Agree	Strongly Agree
ii. I believe that participants involved in the study may experience negative side effects	☐	☐	☐	☐	☐

k. I was worried about the effects the supplements would have having on my child	☐	☐	☐	☐	☐
k. Being involved in the study would mean another thing I had to worry about	☐	☐	☐	☐	☐
l. I believe the study is likely to result in permanent improvement in my child's symptoms	☐	☐	☐	☐	☐
p. I find the researcher is knowledgeable about the topic under study and the study procedures	☐	☐	☐	☐	☐
q. I find the researcher is trustworthy	☐	☐	☐	☐	☐
r. Overall I have a positive reaction to this study	☐	☐	☐	☐	☐
p. I believe that it would be acceptable to use this treatment without a patient's consent	☐	☐	☐	☐	☐
r. I believe it would be acceptable to use this study with individuals who cannot choose treatments for themselves	☐	☐	☐	☐	☐

3. If you were worried about the effects the supplements would have on your child, please describe the effects you were worried about:

____

____

____

____

4a. Are there any other reasons involved in deciding not to participate in this study that you can think of?

Yes ☐ No ☐ (please "X")

b. If yes, please specify:

____

____

____

____

Thankyou for your time and feedback!
