# Supplementary material for: How feasible is nutrition intervention research in eating disorders? Lessons learnt from a pilot parallel randomised controlled trial of tyrosine supplementation in adolescents with anorexia nervosa
Source: J Eat Disord. 2024 Nov 15;12:181. doi: 10.1186/s40337-024-01134-5 (PMC11568674; doi:10.1186/s40337-024-01134-5)
Supplement: Supplementary file 2 — Supplementary Material 2 [file 40337_2024_1134_MOESM2_ESM.pdf]

How feasible is nutrition intervention research in eating disorders? Lessons learnt from a pilot parallel randomised controlled trial of tyrosine supplementation in adolescents with anorexia nervosa

Supplementary Material 2: Additional Clinical Outcomes

Table 6: Perceived acceptability of study components between those consenting and declining RCT Participation (*n*=14)

Acceptability Item	Those Consenting RCT (*n*=6 patients and 7 parents) Frequency (%)	Those Declining RCT (*n*=5 patients and 7 parents) Frequency (%)
**Overall Study Perceptions**		
Overall study acceptability		
Patient	5 (83%)	3 (60%)
Parent	7 (100%)	6 (86%)
Overall positive reaction to study		
Patient	3 (50%)	1 (20%)
Parent	5 (71%)	5 (71%)
**Study Procedures Acceptability**		
Study procedures overall		
Patient	2 (33%)	0 (%)
Parent	7 (100%)	5 (71%)
Using a form of nutrition		
Patient	4 (67%)	2 (40%)
Parent	6 (86%)	6 (86%)
Using tablets		
Patient	2 (33%)	0 (0%)
Parent	3 (43%)	2 (29%)
Collecting blood or urine		
Patient	0 (0%)	0 (0%)
Parent	2 (29%)	2 (29%)
Administering psychological tests		
Patient	3 (50%)	0 (0%)
Parent	6 (86%)	5 (71%)
Belief in effectiveness of treatment		
Patient	2 (33%)	0 (0%)
Parent	2 (29%)	4 (57%)
Belief the study may result in permanent improvement		
Patient	0 (0%)	0 (0%)
Parent	0 (0%)	1 (14%)
Belief of potential discomfort		

Patient	3 (50%)	4 (80%)
Parent	3 (43%)	3 (43%)
Belief of potential negative side effects		
Patient	1 (17%)	2 (40%)
Parent	0 (0%)	3 (43%)
Concern for effects of supplementation		
Patient	2 (33%)	2 (40%)
Parent	1 (14%)	1 (14%)
The study being an additional worry		
Patient	3 (50%)	4 (80%)
Parent	4 (57%)	6 (86%)
**Other Components**		
The researcher appears knowledgeable		
Patient	5 (83%)	3 (60%)
Parent	7 (100%)	6 (86%)
The researcher appears trustworthy		
Patient	6 (100%)	3 (60%)
Parent	7 (100%)	6 (86%)
Using this treatment without consent		
Patient	2 (33%)	1 (20%)
Parent	2 (29%)	0 (0%)
Using this for those who cannot choose treatment for themselves		
Patient	1 (17%)	2 (40%)
Parent	5 (71%)	2 (29%)

Note: patients completing the RCT and the study questionnaire *n*=6; parents whose child completed the RCT and who completed the study questionnaire *n*=7; patients who declined RCT participation, though completed the study questionnaire *n*=5; and parents who declined the RCT participation, though completed the study questionnaire *n*=7. Responses were provided on a 5-point Likert scale from strongly disagree to strongly agree and grouped into a dichotomous variable (agree and strongly agree vs other).

Table 7: Group Baseline Symptom-Based Psychological Tests Results (*n*=7)

Psychological Test	Tyrosine (*n*=3) Median Raw Score (Range) [Converted Score]	Placebo (*n*=4) Median Raw Score (Range) [Converted Score]
Eating Disorders Psychopathology		
Restraint	3.6 (1.4-4.8) †	4.1 (3.2-6.0) †
Eating Concern	3.2 (1.4-3.6) †	3.9 (2.8-5.0) †
Weight Concern	5.0 (3.0-5.4) †	4.8 (3.8-5.0) †
Shape Concern	4.5 (3.1-4.9) †	5.5 (5.4-5.6) †
Global score	4.3 (2.2-4.5) †	4.4 (4.2-5.4) †
Anxiety Symptoms [Standard Score (Percentile)]		
State Anxiety	47 (37-58) [55 (73)]	63 (59-79) [67-74 (94-99)] †
Trait Anxiety	58 (43-59) [66 (94)]	71 (67-73) [78-79 (99)] †
Depressive Symptoms [T Score (Percentile)]		
Negative Mood	5 (5-7) [64 (92-93)]	7 (4-11) [71-75 (>97)] †
	0 (0-2) [43-45 (60-69)]	2 (0-3) [56-64 (87-95)]
Interpersonal Problems		
Ineffectiveness	2 (2-6) [52 (74-76)]	3 (2-5) [53-59 (78-88)]
Anhedonia	5 (1-8) [56-58 (81-82)]	8 (4-13) [66-67 (93)] †
	4 (0-5) [62-64 (91-95)]	3 (2-9) [55-58 (81-89)]
Negative Self- Esteem		
	20 (8-24) [65-66 (92-95)] †	21 (16-40) [62-68 (88-95)] †
Total Score		
Obsessive Compulsive Symptoms		
Compulsions Symp.	28 (28-42)	37 (34-41)
Compulsions Imp.	13 (11-16)	15 (7-19)
Obsessions Symp.	17 (14-24)	23 (17-27)
Obsessions Imp.	14 (9-16)	16 (8-17)
Total Impairment	25 (22-32) †	31 (15-36) †

	Tyrosine		Placebo	
	Patient	Parent	Patient	Parent
Strengths and Difficulties				
Emotional Problems	4 (4-8)	6 (0-6) †	9 (5-10) †	8 (3-9) †
Conduct Problems	1 (0-2)	1 (0-1)	4 (0-4)	3 (2-4)
Hyperactivity	6 (4-6)	7 (5-9) †	2 (0-5)	6 (2-7)
Peer Problems	1 (1-6)	3 (0-4)	3 (1-5)	3 (2-4)
Prosocial Behaviour	10 (8-10)	8 (6-10)	10 (10-10)	10 (6-10)
	11 (10-22)	22 (12-27) †	13 (0-15)	18 (15-20) †
Total Difficulties				
Total Impact	1 (0-2)	2 (0-4) †	5 (1-6) †	5 (3-8) †

Note: † denotes within the clinically significant range, based on 2 standard deviations from the mean or equivalent (1), aside from where test instructions indicated otherwise, including for depressive symptomatology a T score of ≥65 (2) and for obsessive compulsive symptomatology a total impairment score of ≥17 (3) and an abnormal classification for the Strengths and Difficulties Questionnaire (4, 5).

Table 8: Group Comparison of the Baseline Cognitive Performance Tests Results for (n=7)

Cognitive Function Test	Tyrosine (*n*=3) Median Raw Score (Range) [Converted Score]	Placebo (*n*=4) Median Raw Score (Range) [Converted Score]
Acquired Knowledge		
Reading Test [Standard Score]	41 (36-53) [95]	42 (40-43) [98]
Visuospatial Memory		
RCFT Immediate [T Score]	24 (9-31) [51]	23 (16-27) [49]
RCFT Delayed [T Score]	24 (11-30) [51]	25 (16-27) [53]
RCFT Recognition [T Score]	21 (20-22) [51]	21 (19-22) [51]
Visual Learn. Total [Scaled Score]	41 (20-50) [13]	37 (22-41) [12]
Visual Learning Delayed [N/A]	8 (6-13) [N/A]	10 (4-14) [N/A]
Visuospatial Processing		
RCFT Copy Accuracy [N/A]	34 (28-35) [N/A]	35 (33-35) [N/A]
WRAVMA Matching [Standard Score]	38 (29-38) [106]	32 (31-39) [85]
Set Shifting		
Verbal Fluency Switch.[Scaled Score]	13 (12-14) [10]	12 (12-14) [9]
Verb. Fl. Cat. SwitVsCat. Fl.[Scaled Score]	-3 (-5-0) [7]	-2 (-6--1) [8]
Color Word Inh./Swit. vs Inh.[Scaled Score]	-1 (-4-4) [9]	-1 (-2-10) [9]
Trails Condition 4 [Scaled Score]	46 (43-70) [12]	57 (39-93) [11]
Trails Condition 4 all error [Scaled Score]	0.0 (0-0) [12]	0 (0-1) [12]
Design Fluency Condition 3 [Scaled Score]	10 (6-13) [12]	10 (5-11) [12]
Central Coherence Copy		
Order of Construction Index	1.8 (1.0-1.8)	2.0 (1.7-2.7)
Style Index	1.5 (0.7-1.7)	1.3 (1.2-1.5)
Central Coherence Index	1.3 (0.6-1.4)	1.3 (1.2-1.5)
Central Coherence Immediate		
Order of Construction Index	2.6 (2.5-2.6)	2.7 (2.5-3.0)
Style Index	1.5 (1.5-2.0)	1.6 (1.0-2.0)
Central Coherence Index	1.5 (1.5-1.8)	1.6 (1.3-1.9)
Central Coherence Delayed		
Order of Construction Index	2.5 (2.2-3.0)	2.7 (2.5-2.8)
Style Index	1.5 (1.5-2.0)	1.7 (1.0-2.0)
Central Coherence Index	1.7 (1.5-1.7)	1.7 (1.3-1.9)
Verbal Fluency		
Verbal Fluency Letter [Scaled Score]	32 (20-56) [10]	26 (17-31) [9]
Verbal Fluency Category [Scaled Score]	43 (34-51) [12]	39 (36-56) [11]
Verbal Fluency Switching [Scaled Score]	13 (12-14) [10]	12 (12-14) [9]
Planning and Problem Solving		
Tower Total Achievement [Scaled Score]	18 (15-20) [11]	14 (13-19) [8]
Design Fluency Condition 1 [Scaled Score]	12 (9-12) [12]	11 (7-17) [11]
Design Fluency Condition 2 [Scaled Score]	12 (11-14) [11]	14 (8-16) [13]
Design Fluency Composite [Scaled Score]	36 (34-37) [13]	35 (29-42) [12]
Processing Speed		
Digit Symbol Coding [Scaled Score]	73 (54-81) [11]	57 (43-85) [8]
Trails Condition 1 [Scaled Score]	22 (17-28) [10]	17 (13-21) [12]
Trails Condition 2 [Scaled Score]	23 (19-42) [12]	29 (25-41) [11]
Trails Condition 3 [Scaled Score]	26 (22-36) [12]	23 (21-35) [12]

Cognitive Function Test	Tyrosine (*n*=3) Median Raw Score (Range) [Converted Score]	Placebo (*n*=4) Median Raw Score (Range) [Converted Score]
Cognitive Inhibition		
CW Condition 3 [Scaled Score]	64 (34-65) [7]	48 (40-90) [11]
CW Condition 4 [Scaled Score]	56 (47-96) [11]	54 (52-66) [11]
CW InhibVsColor [Scaled Score]	2 (-3-4) [12]	0 (-8-3) [10]
CW InhSwVsComNamRead [Scaled Score]	1 (0-1) [11]	-1 (-2-1) [9]
CW Total Err. Inhib.[Scaled Score]	2 (0-2) [11]	2 (0-4) [11]
CW Total Err. Inhib/Swi. [Scaled Score]	2 (0-7) [11]	2 (0-6) [11]
Verbal Fluency Repetition [Scaled Score]	1 (0-2) [8]	2 (1-4) [7]
Verbal Fluency Set Loss [Scaled Score]	2 (1-2) [10]	2 (0-2) [10]
Verbal Fluency % Set Loss [Scaled Score]	3.4 (2.4-4.3) [9]	1.6 (0-5.3) [10]
Tower Rule Violation [Percentile]	0.0 (0-0) [100.0] †	2 (0-4) [36]
Design Fluency Set Loss [Scaled Score]	1 (0-3) [13]	5 (1-7) [9]
Design Fluency Repeated [Scaled Score]	1 (1-1) [13]	3 (0-7) [12]
Fine Motor Skills		
Trails Condition 5 [Scaled Score]	20 (14-49) [12]	19 (16-24) [12]
Verbal Memory		
Verbal Paired First 3 Easy	12 (11-12) [N/A]	12 (11-12) [N/A]
Verbal Paired First 3 Hard	10 (10-10) [N/A]	8 (7-10) [N/A]
Verbal Paired Delayed Easy	4 (4-4) [N/A]	4 (4-4) [N/A]
Verbal Paired Delayed Hard	4 (3-4) [N/A]	4 (3-4) [N/A]
Speeded Word Naming		
CW Condition 1 [Scaled Score]	29 (23-45) [10]	29 (20-32) [10]
CW Condition 2 [Scaled Score]	23 (19-37) [10]	21 (18-23) [11]
CW Error Color Naming [Percentile]	1 (0-1) [35]	1 (0-4) [35]
CW Error Word Reading [Percentile]	0 (0-1) [100] †	1 (0-2) [20]

Note: † denotes within the clinically significant range, based on 2 standard deviations from the mean or equivalent for cognitive function tests (1).

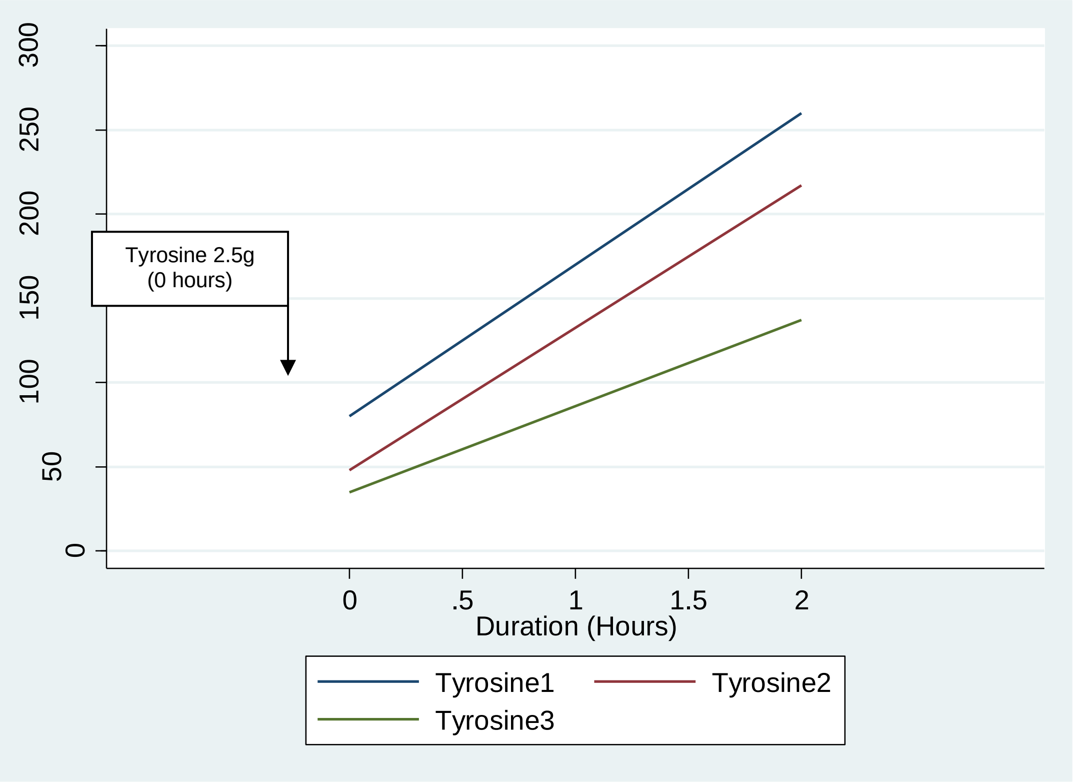

Figure 2: Baseline Blood Tyrosine Response to an Initial Oral Tyrosine Load in Participants Receiving Tyrosine Supplements (*n*=3)

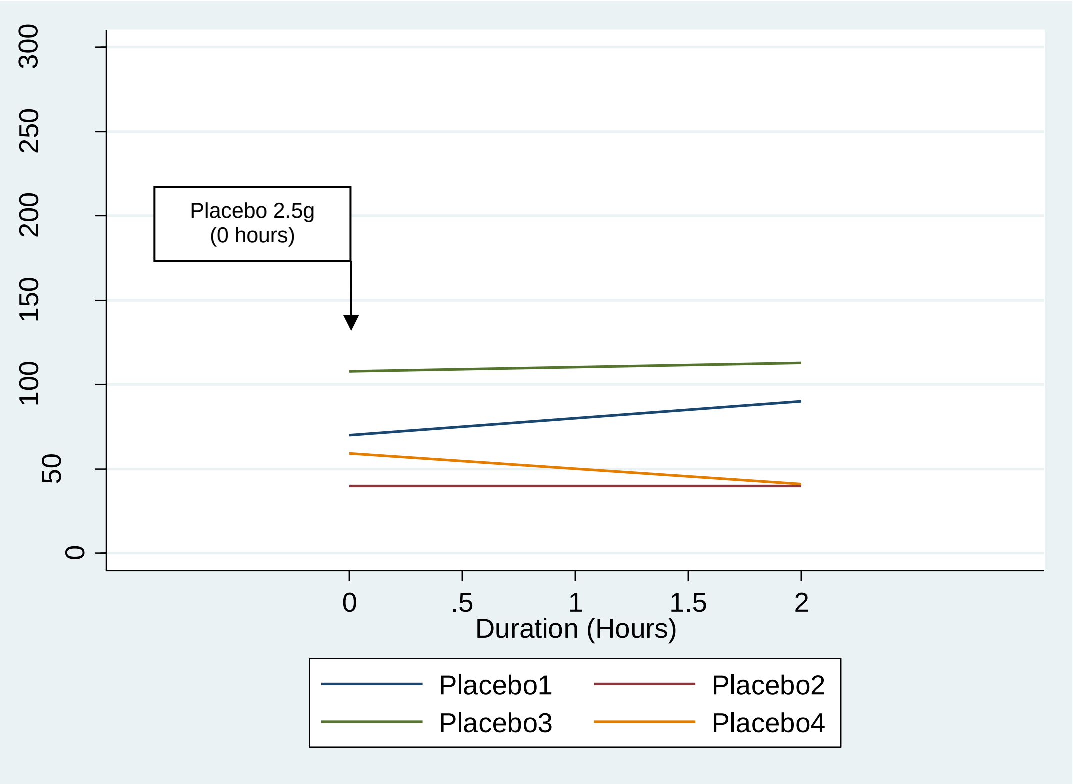

Figure 3: Baseline Blood Tyrosine Response to an Initial Oral Placebo Load in Participants Receiving Placebo Supplements (*n*=4)

Table 9: Comparison of the Median Group Blood Tyrosine Response to Supplementation Over Time (*n*=7)

Time Point (μmol/L)	Blood Tyrosine Time 1 (0 hours)		Blood Tyrosine Time 2 (2 hours)		Absolute Difference
	Median (Range)	*p*-value	Median (Range)	*p*-value	(% Change)
Baseline					
Tyrosine (*n*=3)	48 (35-80)	0.48	217 (137-260) †	0.03*	169 (352%)
Placebo (*n*=4)	65 (40-108)		66 (40-113)		1 (2%)
Week 1					
Tyrosine (*n*=3)	54 (54-149)	0.48	186 (153-249) †	0.03*	132 (244%)
Placebo (*n*=4)	50 (39-94)		55 (31-107)		5 (10%)
Week 6					
Tyrosine (*n*=3)	69 (68-99)	0.83	136 (107-291) †	0.05	67 (97%)
Placebo (*n*=4)	70 (40-111)		76 (45-106)		6 (9%)
Week 12					
Tyrosine (*n*=3)	71 (67-84)	0.16	113 (93-264) †	0.08	42 (59%)
Placebo (*n*=4)	52 (49-77)		47 (23-97)		-5 (-10%)

Note: Absolute Difference denotes the group difference in Time 1 and Time 2 median blood tyrosine; % Change denotes percentage change in group Time 1 and Time 2 median blood tyrosine; † denotes outside of the laboratory reference range.

Table 10: Comparison of Mean Group Creatine-Corrected Urinary Catecholamines and Metabolites at Week 1 of Supplementation (*n*=7)

Measure (mmol/mol creatinine)	Tyrosine Median (Range) (*n*=3)	Placebo Median (Range) (*n*=4)
Dopamine	0.12 (0.04-0.14)	0.15 (0.11-0.22)
Noradrenalin	0.016 (0.016-0.024)	0.026 (0.017-0.032)
Adrenaline	0.005 (0.003-0.01)	0.011 (0.001-0.014)
HVA	2.39 (2.23-4.25)	2.06 (1.57-2.25)
VMA	2.16 (1.28-2.57)	1.09 (0.9-2.27) †
5-HIAA	1.94 (1.93-2.29) †	1.77 (1.32-2.43) †

Note: † denotes outside of the laboratory reference range; HVA denotes homovanillic acid; VMA vanilylmandelic acid; and 5-HIAA denotes 5-Hydroxyindoleacetic acid.

Table 11: Median Group Change in Percent Expected Body Weight Over Time (*n*=7)

Group	Baseline %EBW Median (Range)	Week 6 %EBW Median (Range) [% Change]	Week 12 %EBW Median (Range) [% Change]
Tyrosine (n=3)	85% (84-91)	89% (83-91) [4%]	87% (84-99) [2%]
Placebo (n=4)	81% (80-89)	86% (75-93) [5%]	87% (77-93) [6%]

Note: %EBW denotes percentage expected body weight; % Change denotes the mean percentage change in group %EBW from baseline.

Table 12: Comparison of Median Group Change in State and Trait Anxiety Over Twelve Weeks of Supplementation

Anxiety Symptom Scale	Baseline (*n*=7) Median Raw Score (Range) [Standard Score (Percentile)]	Week 1 (*n*=7) Median Raw Score (Range) [Standard Score (Percentile)]	Week 1 (*n*=7) Absolute Difference (% Change)	Week 6 (*n*=7) Median Raw Score (Range) [Standard Score (Percentile)]	Week 6 (*n*=7) Absolute Difference (% Change)	Week 12 (*n*=6) Median Raw Score (Range) [Standard Score (Percentile)]	Week 12 (*n*=6) Absolute Difference (% Change)
**State Anxiety**							
Tyrosine	47 (37-58) [55 (73)]	39 (32-55) [49 (49)]	-8 (-17%)	40 (31-77) [50 (51)]	-7 (-15%)	44 (42-46) [53 (64)]	-3 (-6%)
Placebo	63 (59-79) [67-74 (94-99)] †	54 (41-66) [60-65 (86-94)] ‡	-9 (-14%)	45 (41-50) [53-56 (68-73)]	-18 (-29%)	49 (42-65) [57-60 (78-85)]	-14 (-22%)
**Trait Anxiety**							
Tyrosine	58 (43-59) [66 (94)]	54 (45-66) [62 (90)]	-4 (-7%)	50 (38-71) [59 (80)]	-8 (-14%)	51 (41-60) [59 (83)]	-7 (-12%)
Placebo	71 (67-73) [78-79 (99)] †	61 (53-73) [69-70 (97-98)] †	-10 (-14%)	59 (44-65) [67-68 (95-97)] ‡	-12 (-17%)	53 (47-71) [61-62 (88-90)]	-18 (-25%)

Note: anxiety scale questions are given a weighted score of 1 to 4 and weighted scores are added to give the scale score; higher scores for the anxiety scales indicates greater anxiety;
Absolute Difference denotes the difference in median group baseline and follow-up raw scores;
% Change denotes percentage change in group median raw score over time;
† denotes clinically significant anxiety symptoms based on 2 or more standard deviations from the mean (T score of 70 or more) (1, 6); and
‡ denotes a clinically significant change (moved into or out of the clinically significant range).

Table 13: Comparison of Median Group Change in Depressive Symptomatology Over Twelve Weeks of Supplementation

Symptom Scale	Baseline (n=7) Median Raw Score (Range) [T Score (Percentile)]	Week 6 (n=7) Median Raw Score (Range) [T Score (Percentile)]	Absolute Difference (% Change)	Week 12 (n=6) Median Raw Score (Range) [T Score (Percentile)]	Absolute Difference (% Change)
Negative Mood					
Tyrosine	5 (5-7) [64 (92-93)]	3 (3-12) [54 (75-82)]	-2 (-40%)	4 (3-4) [59 (86-88)]	-1 (-20%)
Placebo	7 (4-11) [71-75 (>97)] †	6 (5-8) [66-70 (95-97)] †	-1 (-14%)	7 (3-8) [71-75 (>97)] †	0 (0%)
Interpersonal Problems					
Tyrosine	0 (0-2) [43-45 (60-69)]	0 (0-6) [43-45 (60-69)]	0 (N/A)	1 (0-1) [54-56 (83-89)]	1 (N/A)
Placebo	2 (0-3) [56-64 (87-95)]	1 (0-3) [50-54 (78-83)]	-1 (-50%)	2 (0-4) [56-64 (87-95)]	0 (0)
Ineffectiveness					
Tyrosine	2 (2-6) [52 (74-76)]	4 (0-6) [64-66 (91-97)] †‡	2 (100%)	3 (0-6) [58-59 (87-88)] ‡	1 (50%)
Placebo	3 (2-5) [53-59 (78-88)]	3 (1-4) [53-59 (78-88)]	0 (0%)	4 (0-5) [59-66 (88-97)] †‡	1 (33%)
Anhedonia					
Tyrosine	5 (1-8) [56-58 (81-82)]	4 (2-16) [52-54 (87-90)]	-1 (-20%)	4 (2-5) [52-54 (87-90)]	-1 (-20%)
Placebo	8 (4-13) [66-67 (93)] †	7 (2-7) [62-63 (88-90)] ‡	-1 (-13%)	5 (3-7) [55-56 (76-82)]	-3 (-38%)
Negative Self-Esteem					
Tyrosine	4 (0-5) [62-64 (91-95)]	1 (0-9) [45-46 (51-53)]	-3 (-75%)	2 (0-3) [51-52 (74)]	-2 (-50%)
Placebo	3 (2-9) [55-58 (81-89)]	3 (1-5) [55-58 (81-89)]	0 (0%)	3 (0-5) [55-58 (81-89)]	0 (0%)
Total Score					
Tyrosine	20 (8-24) [65-66 (92-95)] †	12 (5-49) [54 (72-76)] ‡	-8 (-40%)	12 (6-18) [54 (72-76)]	-8 (-40%)
Placebo	21 (16-40) [62-68 (88-95)] †	19 (15-20) [59-65 (87-93)] †	-2 (-10)	21 (6-27) [62-68 (88-95)] †	0 (0%)

Note: scale questions consist of three choices (keyed as 0, 1 or 2), keyed scores are added to give the score for each scale and the total score is determined by adding all keyed items; higher scores indicate greater severity;
Absolute Difference denotes the difference in median group baseline and follow-up raw scores;
% Change denotes percentage change in group median raw score over time;
† denotes clinically significant depressive symptomatology, based on criteria defined by Kovacs (2001) (T score of 65 or greater) (2); and
‡ denotes a clinically significant change (moving into or out of the clinically significant range).

Table 14: Comparison of Median Group Change in Obsessive Compulsive Symptomatology Over Twelve Weeks of Supplementation

Symptom Scale	Baseline (n=7) Median Raw Score (Range)	Week 6 (n=7) Median Raw Score (Range)	Absolute Difference (% Change)	Week 12 (n=6) Median Raw Score (Range)	Absolute Difference (% Change)
Compulsions Symptoms					
Tyrosine	28 (28-42)	35 (26-37)	7 (25%)	31 (29-32)	3 (11%)
Placebo	37 (34-41)	37 (33-41)	0 (0%)	37 (31-47)	0 (0%)
Compulsions Impairment					
Tyrosine	13 (11-16)	10 (8-16)	-3 (-23%)	10 (6-13)	-3 (-23%)
Placebo	15 (7-19)	11 (8-16)	-4 (-27%)	15 (5-18)	0 (0%)
Obsessions Symptoms					
Tyrosine	17 (14-24)	16 (14-28)	-1 (-6%)	14 (14-14)	-3 (-18%)
Placebo	23 (17-27)	19 (13-22)	-4 (-17%)	20 (16-26)	-3 (-13%)
Obsessions Impairment					
Tyrosine	14 (9-16)	5 (4-17)	-9 (-64%)	13 (9-16)	-1 (-7%)
Placebo	16 (8-17)	12 (9-19)	-4 (-25%)	14 (7-18)	-2 (-13%)
Total Impairment					
Tyrosine	25 (22-32) †	20 (13-27) †	-5 (-20%)	22 (15-29) †	-3 (-12%)
Placebo	31 (15-36) †	22 (19-35) †	-9 (-29%)	29 (12-36) †	-2 (-6%)

Note: symptom scale questions are scored on a three-point scale and impairment scales are scored on a 5-point scale; scale scores are determined by adding the score for each scale item, while the total impairment score is determined by adding the compulsions impairment and obsessions impairment scores; higher scores indicate greater severity;
Absolute Difference denotes the difference in median group baseline and follow-up raw scores;
% Change denotes percentage change in group median raw score over time; and
† denotes clinically significant total impairment symptoms, based on a Total Impairment score of ≥17 (3).

Table 15: Comparison of Median Group Change in Strengths and Difficulties Questionnaire Results Following Twelve Weeks of Supplementation

Symptom Scale	Baseline (*n*=7) Median Raw Score (Range)	Week 6 (*n*=7) Median Raw Score (Range)	Absolute Difference (% Change)	Week 12 (*n*=6) Median Raw Score (Range)	Absolute Difference (% Change)
**Emotional Problems**					
Patient					
Tyrosine	4 (4-8)	5 (3-7)	1 (25%)	4 (3-5)	0 (0%)
Placebo	9 (5-10) †	7 (4-8) †	-2 (-22%)	8 (5-8) †	-1 (-11%)
Parent					
Tyrosine	6 (0-6) †	6 (0-7) †	0 (0%)	3 (0-6) ‡	-3 (-50%)
Placebo	8 (3-9) †	4 (1-7) ‡	-4 (-50%)	5 (3-5) †‡	-3 (-38%)
**Conduct Problems**					
Patient					
Tyrosine	1 (0-2)	0 (0-5)	-1 (-100%)	0 (0-0)	-1 (-100%)
Placebo	4 (0-4)	5 (3-7) †‡	1 (25%)	5 (2-7) †	1 (25%)
Parent					
Tyrosine	1 (0-1)	1 (0-3)	0 (0%)	0 (0-0)	-1 (-100%)
Placebo	3 (2-4)	3 (1-5)	0 (0%)	2 (0-4)	-1 (-33%)
**Hyperactivity**					
Patient					
Tyrosine	6 (4-6)	5 (3-8)	-1 (-17%)	5 (5-5)	-1 (-17%)
Placebo	7 (5-9) †	7 (2-8) †	0 (0%)	7 (6-10) †	0 (0%)
Parent					
Tyrosine	2 (0-5)	4 (0-5)	2 (100%)	2 (0-4)	0 (0%)
Placebo	6 (2-7)	5 (0-6)	-1 (-17%)	4 (2-5)	-2 (-33%)
**Peer Problems**					
Patient					
Tyrosine	1 (1-6)	2 (0-7)	1 (100%)	1 (0-2)	0 (0%)
Placebo	3 (1-5)	2 (1-4)	-1 (-33%)	2 (2-3)	-1 (-33%)
Parent					
Tyrosine	3 (0-4)	2 (0-3)	-1 (-33%)	2 (0-3)	-1 (-33%)
Placebo	3 (2-4)	2 (0-2)	-1 (-33%)	2 (0-3)	-1 (-33%)

Symptom Scale	Baseline (*n*=7) Median Raw Score (Range)	Week 6 (*n*=7) Median Raw Score (Range)	Absolute Difference (% Change)	Week 12 (*n*=6) Median Raw Score (Range)	Absolute Difference (% Change)
**Prosocial Behaviour**					
Patient					
Tyrosine	10 (8-10)	10 (1-10)	0 (0%)	10 (1-10)	0 (0%)
Placebo	8 (6-10)	8 (6-9)	0 (0%)	7 (6-9)	-1 (-13%)
Parent					
Tyrosine	10 (10-10)	10 (6-10)	0 (0%)	10 (10-10)	0 (0%)
Placebo	10 (6-10)	8 (4-10)	-2 (-20%)	9 (7-10)	-1 (-10%)
**Total Difficulties**					
Patient					
Tyrosine	11 (10-22)	10 (8-27)	-1 (-9%)	10 (8-12)	-1 (-9%)
Placebo	22 (12-27) †	21 (13-23) †	-1 (-5%)	23 (15-26) †	1 (5%)
Parent					
Tyrosine	13 (0-15)	13 (0-18)	0 (0%)	7 (0-13)	-6 (-46%)
Placebo	18 (15-20) †	13 (4-17) ‡	-5 (-28%)	14 (5-16)	-4 (-22%)
**Total Impact**					
Patient					
Tyrosine	1 (0-2)	1 (0-3)	0 (0%)	2 (1-3) †‡	1 (100%)
Placebo	5 (1-6) †	4 (0-6) †	-1 (-20%)	3 (0-7) †	-2 (-40%)
Parent					
Tyrosine	2 (0-4) †	2 (0-6) †	0 (0%)	0 (0-0) ‡	-2 (-100%)
Placebo	5 (3-8) †	2 (0-6) †	-3 (-60%)	0 (0-10) ‡	-5 (100%)

Note: questions for the five subscales are scored on a three-point scale while the impact questions are scored on a 5-point scale; question responses are scored then added to determine scale scores; the total difficulties score is determined by adding the first four subscale scores (excluding prosocial behaviour); for all scores a higher score indicates greater severity, aside from the prosocial scale where a higher score indicates lower severity;

Absolute Difference denotes the difference in median group baseline and follow-up raw scores; % Change denotes percentage change in group median raw score over time;

† denotes within the clinically significant range based on a score within the abnormal range (4, 5); and

‡ denotes a clinically significant change (moving into or out of the clinically significant range).

Table 16: Comparison of Median Group Change in Verbal Fluency and Processing Speed Over the Study

Cognitive Function Test	Baseline (*n*=7) Median Raw Score (Range) [Converted Score]	Week 12 (*n*=6) Median Raw Score (Range) [Converted Score]	Absolute Difference (% Change)	RCI_{PE}	RCI_{EstSRB}
**Verbal Fluency**					
Letter Total [Scaled Score]					
Tyrosine	32 (20-56) [10]	45 (36-54) [13]	13 (41%)	1.06	1.00
Placebo	26 (17-31) [9]	35 (22-42) [11]	9 (35%)	0.59	0.59
Category Total [Scaled Score]					
Tyrosine	43 (34-51) [12]	52 (48-56) [16] †‡	9 (21%)	1.56	1.17
Placebo	39 (36-56) [11]	43 (41-57) [12]	4 (10%)	0.02	-0.14
Switching [Scaled Score]					
Tyrosine	13 (12-14) [10]	17 (14-19) [15]	4 (31%)	1.57	1.79*
Placebo	12 (12-14) [9]	14 (10-16) [12]	2 (17%)	0.73	0.77
**Processing Speed**					
Digit Symbol Coding [Scaled Score]					
Tyrosine	73 (54-81) [11]	83 (77-88) [15]	10 (14%)	2.37*	2.19*
Placebo	57 (43-85) [8]	60 (45-92) [9]	3 (5%)	-0.08	0.17
Trails Condition 1 [Scaled Score]					
Tyrosine	22 (17-28) [10]	19 (18-19) [11]	-3 (-14%)	-0.35	-0.30
Placebo	17 (13-21) [12]	17 (12-20) [12]	0 (0%)	-0.70	-0.37
Trails Condition 2 [Scaled Score]					
Tyrosine	23 (19-42) [12]	21 (18-24) [13]	-2 (-9%)	0.00	0.28
Placebo	29 (25-41) [11]	25 (17-33) [12]	-4 (-14%)	0.00	0.10
Trails Condition 3 [Scaled Score]					
Tyrosine	26 (22-36) [12]	19 (19-19) [14]	-7 (-27%)	0.10	0.25
Placebo	23 (21-35) [12]	20 (19-28) [13]	-3 (-13%)	-0.24	-0.11

Note: an increase in raw score for all tests in this table indicates an improvement in performance for the task, aside from the Trails tasks where an increase indicates a decline; Absolute Difference denotes the difference in median group baseline and follow-up raw scores; % Change denotes percentage change in group median raw score over time; RCI_{PE} denotes reliable change index plus practice effects formula (7); RCI_{EstSRB} denotes reliable change index estimated standardised regression-based formula (8); † denotes within the clinically significant range, based on 2 standard deviations from the mean or equivalent (1); ‡ denotes a clinically significant change (moving into or out of the clinically significant range); and *denotes statistically significant reliable change (RCI=±1.645) (9).

Table 17: Comparison of Median Group Change in Cognitive Inhibition, Planning and Problems Solving and Fine Motor Skill Tasks Following Twelve Weeks of Supplementation

Cognitive Function Test	Baseline (*n*=7) Raw Score (Range) [Converted Score]	Week 12 (*n*=6) Raw Score (Range) [Converted Score]	Absolute Difference (% Change)	RCI_{PE}	RCI_{EstSRB}
**Cognitive Inhibition**					
Color Word Condition 3 [Scaled Score]					
Tyrosine	64 (34-65) [7]	47 (33-60) [11]	-17 (-27%)	1.88*	1.77*
Placebo	48 (40-90) [11]	48 (33-67) [11]	0 (0%)	-1.09	-1.08
Color Word Condition 4 [Scaled Score]					
Tyrosine	56 (47-96) [11]	49 (42-55) [12]	-7 (-13%)	-0.44	-0.49
Placebo	54 (52-66) [11]	51 (40-71) [12]	-3 (-6%)	-0.44	-0.49
Color Word Inhibition vs Color [Scaled Score]					
Tyrosine	2 (-3-4) [12]	-1 (-3-2) [9]	-3 (-150%)	N/A	N/A
Placebo	0 (-8-3) [10]	1 (-4-1) [11]	1 (N/A)	N/A	N/A
Color Word Inhib/Switch vs Comb Naming + Reading [Scaled Score]					
Tyrosine	1 (0-1) [11]	2 (1-2) [12]	1 (100%)	N/A	N/A
Placebo	-1 (-2-1) [9]	0 (-2-3) [10]	1 (100%)	N/A	N/A
Color Word Total Errors Inhibition [Scaled Score]					
Tyrosine	2 (0-2) [11]	2 (0-4) [11]	0 (0%)	N/A	N/A
Placebo	2 (0-4) [11]	2 (0-4) [11]	0 (0%)		
Color Word Total Errors Inhibition/Switch [Scaled Score]					
Tyrosine	2 (0-7) [11]	0 (0-0) [13]	-2 (-100%)	N/A	N/A
Placebo	2 (0-6) [11]	2 (1-2) [11]	0 (0%)	N/A	N/A
Verbal Fluency Repetition [Scaled Score]					
Tyrosine	1 (0-2) [8]	3 (1-4) [7]	2 (200%)	N/A	N/A
Placebo	2 (1-4) [7]	0 (0-1) [10]	-2 (-100%)	N/A	N/A
Verbal Fluency Set Loss [Scaled Score]					
Tyrosine	2 (1-2) [10]	1 (0-1) [11]	-1 (-50%)		N/A
Placebo	2 (0-2) [10]	1 (0-3) [11]	-1 (-50%)	N/A	N/A
Verbal Fluency Percent Set Loss [Scaled Score]					
Tyrosine	3.4 (2.4-4.3) [9]	2.7 (0.8-4.6) [9]	-0.7 (-21%)	N/A	N/A
Placebo	1.6 (0-5.3) [10]	0.5 (0-1.1) [11]	-1.1 (-69%)	N/A	N/A

Cognitive Function Test	Baseline (*n*=7) Raw Score (Range) [Converted Score]	Week 12 (*n*=6) Raw Score (Range) [Converted Score]	Absolute Difference (% Change)	RCI_{PE}	RCI_{EstSRB}
Tower Rule Violation [Percentile]					
Tyrosine	0 (0-0) [100.0] †	1 (0-1) [36] ‡	1 (N/A)	N/A	N/A
Placebo	2 (0-4) [36]	0 (0-0) [100.0] †‡	-2 (-100%)	N/A	N/A
Design Fluency Total Set Loss Designs [Scaled Score]					
Tyrosine	1 (0-3) [13]	1 (0-1) [13]	0 (0%)	N/A	N/A
Placebo	5.0 (1-7) [9]	4 (1-5) [10]	-1 (-20%)	N/A	N/A
Design Fluency Total Repeated Designs [Scaled Score]					
Tyrosine	1 (1-1) [13]	4 (2-5) [11]	3 (300%)	N/A	N/A
Placebo	3 (0-7) [12]	6 (3-15) [10]	3 (100%)	N/A	N/A
**Planning and Problem Solving**					
Tower Total Achievement [Scaled Score]					
Tyrosine	18 (15-20) [11]	17 (15-18) [10]	-1 (-6%)	-0.67	-0.71
Placebo	14 (13-19) [8]	19 (17-20) [11]	5 (36%)	0.62	0.54
Design Fluency Condition 1 [Scaled Score]					
Tyrosine	12 (9-12) [12]	14 (14-14) [14]	2 (17%)	0.20	0.07
Placebo	11 (7-17) [11]	13 (9-14) [13]	2 (18%)	0.20	0.13
Design Fluency Condition 2 [Scaled Score]					
Tyrosine	12 (11-14) [11]	13 (11-15) [12]	1 (8%)	-0.21	-0.18
Placebo	14 (8-16) [13]	15 (13-16) [13]	1 (7%)	-0.48	-0.43
Design Fluency Composite [Scaled Score]					
Tyrosine	36 (34-37) [13]	38 (36-40) [13]	2 (6%)	N/A	N/A
Placebo	35 (29-42) [12]	39 (32-43) [14]	4 (11%)	N/A	N/A
**Fine Motor Skills**					
Trails Condition 5 [Scaled Score]					
Tyrosine	20 (14-49) [12]	20 (19-20) [12]	0 (0%)	-0.22	-0.17
Placebo	19 (16-24) [12]	17 (15-22) [13]	-2 (-11%)	0.39	0.47

Note: an increase in raw score for the Cognitive Inhibition and Fine Motor tasks indicates a decline in performance, while for Planning and Problem Solving Tasks, an increase indicates improvement; the raw score for the Color Word Inhibition vs Color and the Color Word Inhib/Switch vs Comb Naming + Reading denotes a scaled score difference; Absolute Difference denotes the difference in median group baseline and follow-up raw scores; % Change denotes percentage change in group median raw score over time; RCI_{PE} denotes reliable change index plus practice effects formula (7); RCI_{EstSRB} denotes reliable change index estimated standardised regression-based formula (8); † denotes within the clinically significant range, based on 2 standard deviations from the mean or equivalent (1); ‡ denotes a clinically significant change (moving into or out of the clinically significant range); and *denotes statistically significant reliable change (RCI=±1.645) (9).

Table 18: Comparison of Median Group Change in Central Coherence Following Twelve Weeks of Supplementation

Cognitive Function Test	Baseline Median Raw Score (Range)	Week 12 Median Raw Score (Range)	Absolute Difference (% Change)
**Copy**			
Order of Construction Index			
Tyrosine	1.8 (1.0-1.8)	2.1 (1.5-2.7)	0.3 (17%)
Placebo	2.0 (1.7-2.7)	2.4 (2.2-2.5)	0.4 (20%)
Style Index			
Tyrosine	1.5 (0.7-1.7)	3.4 (0.8-6.0)	1.9 (127%)
Placebo	1.3 (1.2-1.5)	1.3 (1.0-1.5)	0 (0%)
Central Coherence Index			
Tyrosine	1.3 (0.6-1.4)	1.3 (0.9-1.8)	0 (0%)
Placebo	1.3 (1.2-1.5)	1.4 (1.2-1.5)	0.1 (8%)
**Immediate Recall**			
Order of Construction Index			
Tyrosine	2.6 (2.5-2.6)	2.2 (2.0-2.3)	-0.4 (-15%)
Placebo	2.7 (2.5-3.0)	3.1 (2.0-3.7)	0.4 (15%)
Style Index			
Tyrosine	1.5 (1.5-2.0)	1.5 (1.0-2.0)	0 (0%)
Placebo	1.6 (1.0-2.0)	1.7 (1.3-2.0)	0.1 (6%)
Central Coherence Index			
Tyrosine	1.5 (1.5-1.8)	1.4 (1.1-1.7)	-0.1 (-7%)
Placebo	1.6 (1.3-1.9)	1.7 (1.4-2.1)	0.1 (6%)
**Delayed Recall**			
Order of Construction Index			
Tyrosine	2.5 (2.2-3.0)	2.2 (2.0-2.3)	-0.3 (-12%)
Placebo	2.7 (2.5-2.8)	2.4 (2.0-3.0)	-0.3 (-11%)
Style Index			
Tyrosine	1.5 (1.5-2.0)	1.8 (1.5-2.0)	0.3 (20%)
Placebo	1.7 (1.0-2.0)	1.8 (1.4-2.0)	0.1 (6%)
Central Coherence Index			
Tyrosine	1.7 (1.5-1.7)	1.5 (1.5-1.6)	-0.2 (-12%)
Placebo	1.7 (1.3-1.9)	1.6 (1.4-1.9)	-0.1 (-6%)

Note: an increase in raw score for tests in this table indicates an improvement in performance for the task; Absolute Difference denotes the difference in median group baseline and follow-up raw scores; % Change denotes percentage change in group median raw score over time.

Table 19: Comparison of Median Group Change in Visuospatial Performance Following Twelve Weeks of Supplementation

Cognitive Function Test	Baseline (*n*=7) Raw Score (Range) [Converted Score]	Week 12 (*n*=6) Raw Score (Range) [Converted Score]	Absolute Difference (% Change)	RCI_{PE}	RCI_{EstSRB}
**Visuospatial Memory**					
RCFT Immediate Recall [T Score]					
Tyrosine	24 (9-31) [51]	26 (17-35) [55]	2 (8%)	N/A	N/A
Placebo	23 (16-27) [49]	27 (12-30) [57]	4 (17%)	N/A	N/A
RCFT Delayed Recall [T Score]					
Tyrosine	24 (11-30) [51]	24 (20-29) [51]	0 (0%)	N/A	N/A
Placebo	25 (16-27) [53]	26 (14-26) [55]	1 (4%)	N/A	N/A
RCFT Recognition [T Score]					
Tyrosine	21 (20-22) [51]	21 (21-21) [51]	0 (0%)	N/A	N/A
Placebo	21 (19-22) [51]	22 (15-22) [57]	1 (5%)	N/A	N/A
Visual Learning Total of Learning [Scaled Score]					
Tyrosine	41 (20-50) [13]	40 (26-54) [13]	-1 (-2%)	N/A	N/A
Placebo	37 (22-41) [12]	39 (18-50) [13]	2 (5%)	N/A	N/A
Visual Learning Delayed [N/A]					
Tyrosine	8 (6-13) [N/A]	11 (8-14) [N/A]	3 (38%)	N/A	N/A
Placebo	10 (4-14) [N/A]	12 (2-14) [N/A]	2 (20%)	N/A	N/A
**Visuospatial Processing**					
RCFT Copy Accuracy [N/A]					
Tyrosine	34 (28-35) [N/A]	32 (29-35) [N/A]	-2 (-6%)	N/A	N/A
Placebo	35 (33-35) [N/A]	34 (26-36) [N/A]	-1 (-3%)	N/A	N/A
WRAVMA Matching [Standard Score]					
Tyrosine	38 (29-38) [106]	40 (39-40) [113]	2 (5%)	0.72	0.64
Placebo	32 (31-39) [85]	33 (30-37) [89]	1 (3%)	0.33	0.47

Note: an increase in score for tests in this table indicates improvement;
Absolute Difference denotes the difference in median group baseline and follow-up raw scores;
% Change denotes percentage change in group median raw score over time;
RCI_{PE} denotes RCI plus practice effects formula (7); and
RCI_{EstSRB} denotes RCI estimated standardised regression-based formula (8).

Table 20: Comparison of Median Group Change in Verbal Memory and Speeded Word Naming Over the Study

Cognitive Function Test	Baseline (*n*=7) Raw Score (Range) [Converted Score]	Week 12 (*n*=6) Raw Score (Range) [Converted Score]	Absolute Difference (% Change)	RCI_{PE}	RCI_{EstSRB}
**Verbal Memory**					
Verbal Paired First 3 trials Easy					
Tyrosine	12 (11-12) [N/A]	12 (11-12) [N/A]	0 (0%)	N/A	N/A
Placebo	12 (11-12) [N/A]	12 (12-12) [N/A]	0 (0%)	N/A	N/A
Verbal Paired First 3 trials Hard					
Tyrosine	10 (10-10) [N/A]	12 (12-12) [N/A]	2 (20%)	N/A	N/A
Placebo	8 (7-10) [N/A]	11 (10-12) [N/A]	3 (38%)	N/A	N/A
Verbal Paired Delayed Easy					
Tyrosine	4 (4-4) [N/A]	4 (4-4) [N/A]	0 (0%)	N/A	N/A
Placebo	4 (4-4) [N/A]	4 (4-4) [N/A]	0.0 (0%)	N/A	N/A
Verbal Paired Delayed Hard					
Tyrosine	4 (3-4) [N/A]	4 (4-4) [N/A]	0 (0%)	N/A	N/A
Placebo	4 (3-4) [N/A]	4 (3-4) [N/A]	0 (0%)	N/A	N/A
**Speeded Word Naming**					
Color Word Cdn1 [Scaled Score]					
Tyrosine	29 (23-45) [10]	25 (23-27) [11]	-4 (-14%)	-0.05	-0.05
Placebo	29 (20-32) [10]	26 (20-32) [11]	-3 (-10%)	-0.05	-0.05
Color Word Cdn 2 [Scaled Score]					
Tyrosine	23 (19-37) [10]	23 (19-26) [10]	0 (0%)	0.00	0.01
Placebo	21 (18-23) [11]	22 (19-23) [10]	1 (5%)	-0.52	-0.58
Color Word Total Errors Color Naming [Percentile]					
Tyrosine	1 (0-1) [35]	0 (0-0) [100] †‡	-1 (-100%)	N/A	N/A
Placebo	1 (0-4) [35]	0 (0-3) [100] †‡	-1 (-100%)	N/A	N/A
Color Word Total Errors Word Reading [Percentile]					
Tyrosine	0 (0-1) [100] †	1 (0-1) [20] ‡	1 (N/A)	N/A	N/A
Placebo	1 (0-2) [20]	0 (0-1) [100] †‡	-1 (-100%)	N/A	N/A

Note: an increase in raw score for the Verbal Memory tests in this table indicates an improvement in performance, while for the Speeded Word Naming increase indicates a decline; Absolute Difference denotes difference in median baseline and follow-up raw scores; % Change denotes percentage change in group median raw score over time; RCI_{PE} denotes reliable change index plus practice effects formula (7); RCI_{EstSRB} denotes reliable change index estimated standardised regression-based formula (8); † denotes within the clinically significant range, based on 2 standard deviations from the mean or equivalent (1); and ‡ denotes a clinically significant change (moving into or out of the clinically significant range).

21: Comparison of Median Group Change in Set-Shifting Following Twelve Weeks of Supplementation

Cognitive Function Test	Baseline (*n*=7) Raw Score (Range) [Converted Score]	Week 12 (*n*=6) Raw Score (Range) [Converted Score]	Absolute Difference (% Change)	RCI_{PE}	RCI_{EstSRB}
Verbal Fluency Switching [Scaled Score]					
Tyrosine	13 (12-14) [10]	17 (14-19) [14]	4 (31%)	1.23	1.16
Placebo	12 (12-14) [9]	14 (10-16) [12]	2 (17%)	0.85	0.86
Verbal Fluency Cat Switch vs Category Fluency [Scaled Score]					
Tyrosine	-3 (-5-0) [7]	-2 (-7-3) [8]	1 (33%)	N/A	N/A
Placebo	-2 (-6- -1) [8]	-2 (-10-1) [8]	0 (0%)	N/A	N/A
Color Word Inhibition/Switching vs Inhibition [Scaled Score]					
Tyrosine	-1 (-4-4) [9]	2 (0-3) [12]	3 (300%)	N/A	N/A
Placebo	-1 (-2-10) [9]	0 (-2-4) [10]	1 (100%)	N/A	N/A
Trails Condition 4 [Scaled Score]					
Tyrosine	46 (43-70) [12]	53 (47-58) [11]	7 (15%)	-0.58	-0.59
Placebo	57 (39-93) [11]	60 (47-87) [10]	3 (5%)	-0.58	-0.58
Trails Condition 4 all error types [Scaled Score]					
Tyrosine	0 (0-0) [12]	2 (0-3) [9]	2 (N/A)	N/A	N/A
Placebo	0 (0-1) [12]	1 (0-2) [10]	1 (N/A)	N/A	N/A
Design Fluency Condition 3 [Scaled Score]					
Tyrosine	10 (6-13) [12]	12 (11-12) [14]	2 (20%)	-0.07	-0.13
Placebo	10 (5-11) [12]	11 (8-13) [13]	1 (10%)	-0.36	-0.41

Note: an increase in raw score for tests in this table indicates an improvement in performance for the task, aside from the Trails tasks where an increase indicates a decline; the raw score for the Verbal Fluency Cat Switch vs Category Fluency and the Color Word Inhibition/Switching vs Inhibition denotes a scaled score difference;
Absolute Difference denotes the difference in median group baseline and follow-up raw scores;
% Change denotes percentage change in group median raw score over time;
RCI_{PE} denotes reliable change index plus practice effects formula (7); and
RCI_{EstSRB} denotes reliable change index estimated standardised regression-based formula (8).

References

1. Gregory RJ. Norms and Reliability. Psychological Testing, History, Principles, and Applications, Sixth Edition. Boston: Allyn and Bacon; 2011. p. 67-86.
2. Kovacs M. Children's Depression Inventory Manual. New York: Multi-Health Systems Inc.
3. Shafran R, Frampton I, Heyman I, Reynolds M, Teachman B, S. R. The Preliminary Development of a New Self-Report Measure for OCD in Young People. Journal of Adolescence. 2003;26:137-42.
4. NSW Department of Health. Your Guide to MH-OAT, Clinicians' Reference Guide to NSW Mental Health Outcomes and Assessment Tools. Gladesville: NSW Department of Health; 2001.
5. Goodman R. The Extended Version of the Strengths and Difficulties Questionnaire as a Guide to Child Psychiatric Caseness and Consequent Burden. Journal of Child Psychology and Psychiatry. 1999;40(5):791-9.
6. Dancey C, Reidy J. Statistics without Maths for Psychology: Using SPSS for Windows. London: Prentice-Hall; 2008.
7. Chelune GJ, Naugle RI, Luders H, J. S, Awad IA. Individual Change After Epilepsy Surgery: Practice Effects and Base-Rate Information. Neuropsychology. 1993;7(1):41-52.
8. Maassen GH, Bossema ER, Brand N. Reliable Change Assessment with Practice Effects in Sport Concussion Research: a Comment on Hinton-Bayre. British Journal of Sports Medicine. 2006;40(10):829–33.
9. Duff K. Evidence-Based Indicators of Neuropsychological Change in the Individual Patient: Relevant Concepts and Methods. Archives of Clinical Neuropsychology. 2012;27:248–61.
